# Assessment of escape from X chromosome inactivation and gene expression in single human immune cells

**DOI:** 10.1016/j.xpro.2021.100641

**Published:** 2021-07-20

**Authors:** Sven Hendrik Hagen, Jana Hennesen, Marcus Altfeld

**Affiliations:** 1 Research Department Virus Immunology, Heinrich Pette Institute, Leibniz Institute for Experimental Virology, Martinistrasse 52, Hamburg 20251, Germany; 2 Technology Platform Flow Cytometry / FACS, Heinrich Pette Institute, Leibniz Institute for Experimental Virology, Martinistrasse 52, Hamburg 20251, Germany

**Keywords:** Flow Cytometry/Mass Cytometry, Immunology, Molecular Biology, Gene Expression

## Abstract

X-chromosomal genes escaping from X chromosome inactivation (XCI) in immune cells can contribute to sex-specific differences in immune responses. This protocol describes the specific steps to determine escape from XCI and to simultaneously quantify mRNA expression of multiple genes at the single immune cell level using a single-nucleotide polymorphism approach. The protocol furthermore allows the analysis of allele-specific expression of X-chromosomal genes.

For complete details on the use and execution of this protocol, please refer to Hagen et al. (2020).

## Highlights

• Approach to investigate escape from XCI and gene expression in single cells
• Simultaneous gene expression measurement of over 100 genes in one cell
• Assessment of allele-specific expression of genes with monoallelic expression pattern

## Graphical abstract

**Figure:**
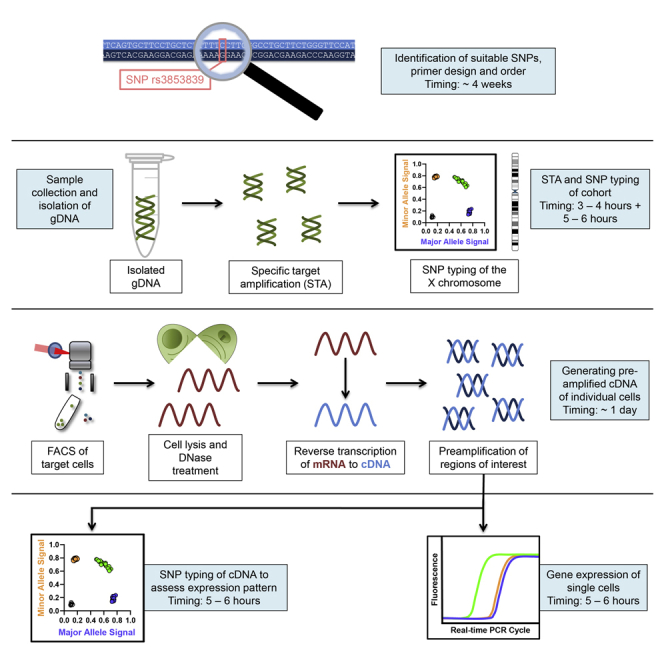

## Before you begin

This protocol describes the specific steps to investigate escape of X-chromosomal genes from X chromosome inactivation (XCI) in human plasmacytoid dendritic cells (pDCs), with the example of *TLR7* and *CYBB*. This protocol can be adapted to investigate the escape of different genes in other cell types. We have also investigated the escape of *RPS6KA3, BTK* and *IL13RA1* from XCI in human pDCs (Hagen et al., 2020).

For X chromosomal genes it is also possible to compare the effect of the minor allele in comparison to the major allele at the single-cell level, for example whether the single-nucleotide polymorphism (SNP) has an influence on the mRNA transcription level. The vast majority of autosomal genes however are expressed in a biallelic fashion. Thus this approach is only feasible for allosomal genes and the roughly 5% of autosomal genes that are randomly expressed in a monoallelic fashion (Gimelbrant et al., 2007).

The first step is to identify adequate SNPs in the gene(s) of interest and to design the specific primers, before SNP typing the cohort of interest to identify female individuals with a suitable SNP profile.

For this experimental approach there are multiple primers employed. The Delta Gene forward and reverse primers are used for preamplification and gene expression analysis. The Delta Gene primers are primers without any modification and the Delta Gene forward and reverse primers are provided as equimolarly pooled at 100 μM.

**Figure 1:**
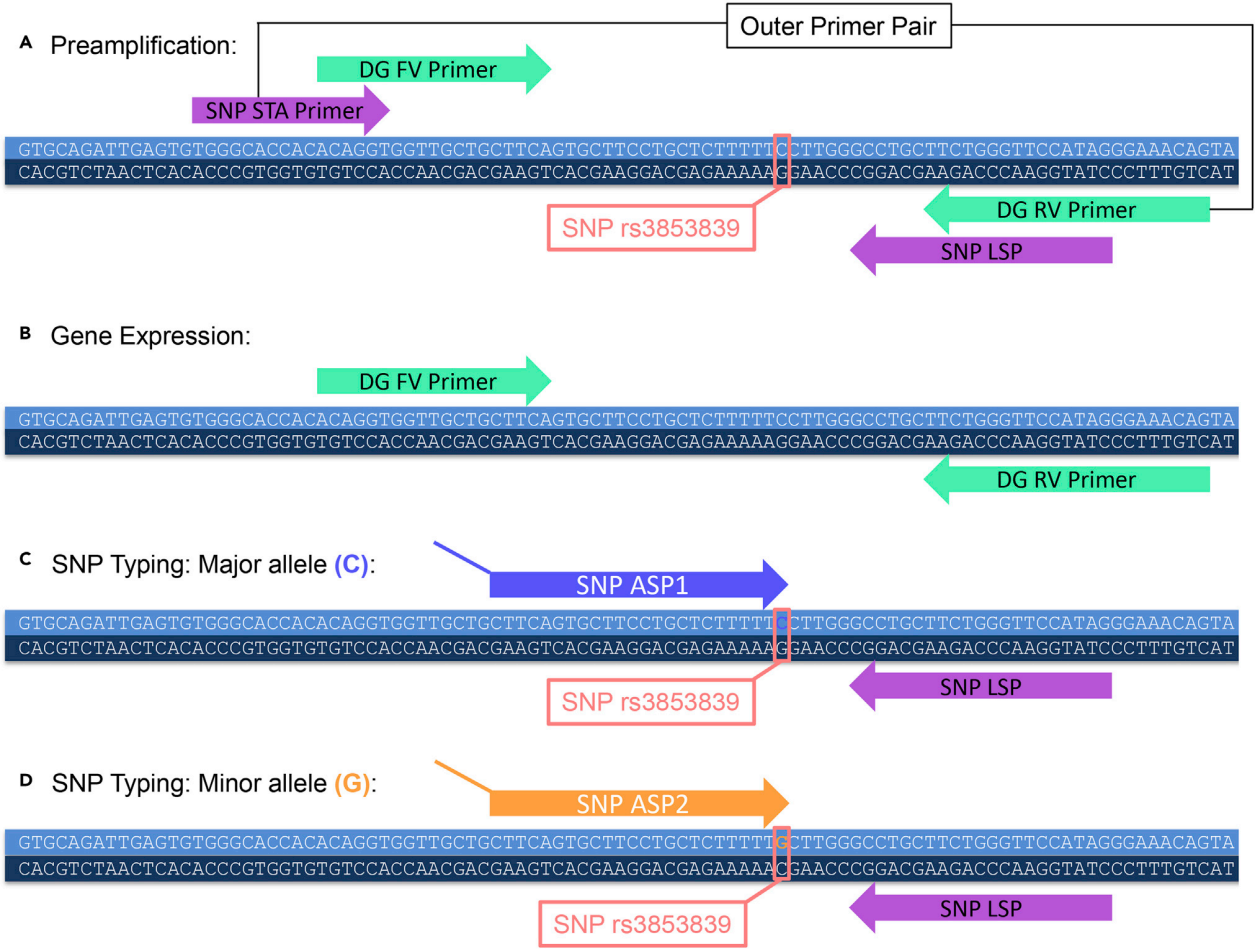
The sequence of the TLR7 transcript surrounding the location of SNP rs3853839 is displayed The transcript information was obtained from the Ensembl Website (Yates et al., 2019). The Figure was adapted from a sequence and primer alignment that was done in CLC Main Workbench (Version 7.9.1). (A) The SNP Type primers (STA primer and LSP) and the Delta Gene primers are aligned and the outer most primer pair is identified for the preamplification step. (B) The Delta Gene primers are used for gene expression analysis. (C and D) (C) ASP1 detects the major allele (C) and (D) ASP2 detects the minor allele (G) during the SNP Typing. If there is no splicing site in the sequence (which is the case for the displayed TLR7 sequence) the same SNP Type primers can be employed for SNP typing of mRNA and gDNA. DG = Delta Gene; STA = specific target amplification; LSP = locus-specific primer; ASP = allele-specific primer; FV = forward; RV = reverse, SNP = single-nucleotide polymorphism.

The SNP Type primers consist of the specific target amplification (STA) primer, the Locus-specific primer (LSP) and the Allele-specific primer (ASP)1 and ASP2. The LSP and STA primer are primers without any modifications. They are individually resuspended at 100 μM. ASP1 and ASP2 are the only primers with specific modifications, hence they cannot be ordered through a different company. ASP1 binds to one allele and ASP2 binds to the other allele (see Figures 1C and 1D). ASP1 and ASP2 possess specific tags. A probe conjugated to FAM is specific for the ASP1 tag, and a probe conjugated to HEX is specific for the ASP2 tag. The LSP and STA primers are used for preamplification of the region of interest and LSP in combination with the ASP1/ASP2 for detection of the different alleles. If there is no splicing site in the proximity of the binding of ASP1/ASP2, LSP or STA primer (meaning that the sequence of the cDNA is identical to the sequence of the genomic DNA (gDNA)) the same primers can be used for SNP Typing of the mRNA and gDNA.

Since during the preamplification step in the C1 (see graphical abstract) an amplicon needs to be generated that can subsequently be analyzed with the Delta Gene and the SNP Type primers, the outer most primer pair of the Delta Gene forward and reverse primer and the LSP and STA primer needs to be used. Figure 1A shows the sequence of TLR7 surrounding the SNP rs3853839 with the binding sites of the Delta Gene primers and the SNP Type primers, the LSP and STA primer. For the preamplification the outer most primer pair is used, which in this case represents the SNP Type STA primer and the Delta Gene reverse primer. As the Delta Gene primers are provided as forward and reverse primer equimolarly pooled, the Delta Gene reverse primer needs to be ordered either individually through a different company or as a separate Delta Gene primer pair (that is only used for the pre-amplification of the TLR7 rs3853839 region) consisting of SNP Type STA primer and the Delta Gene reverse primer.

Figure 1B shows the Delta Gene Primers that are used for the gene expression analysis. Figures 1C and 1D show the SNP typing with ASP1 binding to the major allele (C) and ASP2 binding to the minor allele (G) in combination with the SNP Type primer LSP.

**CRITICAL:** The following steps are crucial, since it is essential to identify donors with suitable SNPs.

### Identification of suitable SNPs and primer design

**Timing: Researching SNPs and designing primers: 1 day**

**Timing: Delivery of primers after ordering: ~4 weeks**

1. Research suitable SNPs for your gene of interest
  It is possible to use NCBI, Ensembl or comparable resources to research SNPs and the sequences of different genes. In the following section we will demonstrate it with the Ensembl website (Yates et al., 2019).
  a. Go to https://www.ensembl.org/index.html
  b. Enter your gene of interest e.g., *TLR7* (we will present the search for an appropriate SNP with the example of *TLR7*).
  c. Select TLR7 (Human Gene).
  d. On the left hand side click on “variant table.” In the variant table focus on the class “SNP”.
  e. Adjust “consequences” to exclude “intron variant” (the SNPs need to be present in the mature mRNA, thus either in the 5′ untranslated region (UTR), the coding sequence or the 3′ UTR).
  f. Adjust the global minor allele frequency (MAF), e.g., to 0.01–0.5 (1%–50%). This also depends on the size of the cohort that you will be working with.
  g. Click on the variant ID and then select population genetics. Verify the MAF of your SNP in the region of the world where your cohort is based. E.g. the SNP rs5741881 in *TLR7* has a worldwide MAF of 6.8%, however the MAF in Europe is 0%.
  h. Also pay attention to the location / consequence of your SNP. As the SNPs are only used as a mean to differentiate between the mRNA of the two X chromosomes, ideally, they do not have a consequence on the transcription or translation of the corresponding protein. Based on the worldwide MAF the second highest SNP in the mature mRNA of TLR7, the SNP rs179008, creates a missense variant with an amino acid exchange in the TLR7 protein. The SNP rs3853839 has a worldwide MAF of 40.2% and a European MAF of 17.1% and is located in the 3′ UTR, so that it does not affect the amino acid sequence of the TLR7 protein.

2. Design the primers for qPCR (Delta Gene) and for the detection of the SNPs (SNP Type primers)
  Refer to the *D3 Assay Design* user guide (100–6812 Rev 06) for more information:
  https://www.fluidigm.com/binaries/content/documents/fluidigm/resources/d3_assay_design_ug_100-6812/d3_assay_design_ug_100-6812/fluidigm%3Afile
  a. Go to the Website D3 Assay Design from Fluidigm and create a log-in:
    http://d3.fluidigm.com/account/login
  b. To design and order the SNP Type primer, click “+New Panel”, select “SNP Type Assays”, select “New Panel”, chose “Homo sapiens (hg38 – UCSC)”, chose “dbSNP150 with >= 1% frequency” and then name your Panel.
  c. For the method for target entry choose target sequence.
  d. To obtain the target sequence go to the Ensemble Website:
    https://www.ensembl.org/index.html.
  e. Enter your gene of interest (e.g., TLR7) and then extract the transcript sequence for the gene of interest (note: if a gene has a lot of different annotated transcripts it is possible to obtain an RNASeq data set of the target cells to investigate the expression levels of the different transcript variants). TLR7 has only one protein coding transcript.
  f. Import the sequence into a program for sequence analysis, e.g., CLC workbench.
  g. For the design of the SNP Type primers, annotate the SNP in the software and then copy the SNP and 50–60 nucleotides up- and downstream of the SNP. Annotate the SNP with the major and the minor nucleotide within brackets: [C/G]
  h. In the D3 Assay Design click “Target Sequence” and then enter the information, e.g.,
    i name: TLR7_rs3853839
    ii sequence: GCAGATTGAGTGTGGGCACCACACAGGTGGTTGCTGCTTCAGTGCTTCCTGCTCTTTTT**[C/G]**CTTGGGCCTGCTTCTGGGTTCCATAGGGAAACAGTAAGAAAGAAAGACACATCCTTACCAT
    iii click "add targets".
    iv repeat for all the primers you would like to design.
    v select your primers and click “Submit for Design”.
  i. If the sequence covers an exon gap, you will need to design a different set of primers for the SNP typing of your cohort. Enter your SNP via the rs ID (e.g., rs3853839).
  j. If the sequence does not cover an exon gap you can use the same primers for the SNP typing of the gDNA and the mRNA (which is the case for the TLR7 SNP rs3853839).
  k. To design and order the Delta Gene primer, click “+New Panel”, select “Delta Gene Assays”, select “New Panel”, chose “Homo sapiens (hg38 – UCSC)”, chose “dbSNP150 with >= 1% frequency” and then name your Panel.
  l. For the Delta Gene primers insert the sequence and replace the SNP Nucleotide by the letter N (indicating any nucleotide) (refer to steps e-h on how to obtain the sequence).
  m. Click “Target Sequence” and then enter the information, e.g.,
    i Name: TLR7_rs3853839_dg
    ii sequence:
    GCAGATTGAGTGTGGGCACCACACAGGTGGTTGCTGCTTCAGTGCTTCCTGCTCTTTTT**N**CTTGGGCCTGCTTCTGGGTTCCATAGGGAAACAGTAAGAAAGAAAGACACATCCTTACCAT
    iii click "add targets".
    iv repeat for all the primers you would like to design.
    v select your primers and click “Submit for Design”.
  n. Once you have ordered your primers align the Delta Gene and SNP Type primers to the sequence. For the primer pool of the pre-amplification mix the outer primer pair is needed. If one of the primers is a Delta Gene primer, order them separately through a different company as Fluidigm supplies the Delta Gene primers forward and reverse primer equimolar pooled. Refer to Figure 1 and the section *Before You Begin* for clarification. For the region of the SNP rs3853839 in *TLR7* the STA primer of the SNP Type primer and the reverse primer of the Delta Gene primers are used for the pre-amplification step, so that the reverse primer of the Delta Gene primers needs to be ordered individually.

### Isolation of gDNA and SNP typing of cohort

**Timing: Extraction of gDNA for up to 10 donors takes 60 min for step 3.**

**Timing: 3–4 h for step 4**

**Timing: 5–6 h for step 5**

3. Isolate gDNA of your cohort
  a. We used the DNeasy Blood & Tissue Kit (Qiagen) to extract gDNA from either 3–5**×**10^6^ freshly isolated PBMCs, 3–5**×**10^6^ PBMCs that were frozen as a pellet at −80°C or from 3 mL of EDTA blood that was frozen at −80°C and processed as following:
  b. If EDTA blood is used, lyse the erythrocytes with 3 mL of ACK lysis buffer for 3 min and then wash with PBS (7 min / 500 g). Repeat for multiple rounds until the pellet is not red anymore. Then use this pellet in the same way according to the manufacturer’s instructions:
    https://www.qiagen.com/lu/resources/resourcedetail?id=68f29296-5a9f-40fa-8b3d-1c148d0b3030&lang=en
  c. The gDNA was eluted in two separate Eppendorf tubes with 35–50 μL of AE buffer and then measured at a NanoDrop. Concentrations of over 100 ng/μL were subsequently used.
4. Specific Target amplification (STA) of gDNA

A specific Target Amplification (STA) of the gDNA was done prior to the SNP typing. A minimum concentration of 100 ng/μL of gDNA was used. The STA was performed according to the *SNP Genotyping* user guide (PN 68000098 Rev.18), p.131ff.

https://www.fluidigm.com/binaries/content/documents/fluidigm/resources/snp-gt-analysis-ug-68000098/snp-gt-analysis-ug-68000098/fluidigm%3Afile

The LSP and STA primers were used for the STA. If there is no splicing site surrounding the SNP, the same primers can be used for the analysis of the gDNA and the mRNA.

5. SNP Typing of the gDNA

There are different formats for the SNP typing analysis. For SNP typing of the gDNA we have used the 48.48 IFC for SNP Typing (analyzing 48 SNPs in 48 gDNAs) and the 192.24 IFC for SNP Typing (analyzing 24 SNPs in 192 gDNAs).

For the 192.24 format refer to the Quick Reference Guide:

*Genotyping with the 192.24 IFC Using SNP Type Assays* (PN 100–3913 C1):

https://www.fluidigm.com/binaries/content/documents/fluidigm/resources/192.24-gt-snp%E2%80%90type-qr-100%E2%80%903913/192.24-gt-snp%E2%80%90type-qr-100%E2%80%903913/fluidigm%3Afile

For the 48.48 format refer to the Quick Reference Guide:

*Genotyping with the 48.48 IFC Using SNP Type Assays* (PN 100–3910 C1):

https://www.fluidigm.com/binaries/content/documents/fluidigm/resources/48.48-gt-snp%E2%80%90type-qr-100%E2%80%903910/48.48-gt-snp%E2%80%90type-qr-100%E2%80%903910/fluidigm%3Afile

***Note:*** For a step to step description see SNP Typing of the mRNA with the 192.24 IFC using SNP Type Assays below.

### Determining cell size and buoyancy

**Timing: 1 h for step 6**

**Timing: 30–45 min for step 7**

6. Determine the size of your cell type of interest

The Fluidigm C1 Single-Cell Preamp IFCs are optimized for different cells sizes. The following sizes are available: 5–10 μm, 10–17 μm, or 17–25 μm. Measure your cell size to estimate the right IFC size to use. For human pDCs we have used the 5–10 μm IFC. Refer to *The single-cell preparation guide* for more information:

https://www.fluidigm.com/binaries/content/documents/fluidigm/marketing/single-cell-preparation-guide-ebook/single-cell-preparation-guide-ebook/fluidigm%3Afile

7. Determining the buoyancy of your cell type of interest
  a. During the C1 run the cell suspension will be mixed with the Suspension Reagent (Fluidigm) to adjust the buoyancy of the cells in the suspension so that the cells are optimally distributed throughout the suspension.
  b. The buoyancy can be adjusted by changing the ratio of the Suspension Reagent to the cell suspension. Refer to *The single-cell preparation guide* for more information:
    https://www.fluidigm.com/binaries/content/documents/fluidigm/marketing/single-cell-preparation-guide-ebook/single-cell-preparation-guide-ebook/fluidigm%3Afile

### Aliquot preparation

**Timing: 1 h for step 8**

**Timing: 10 min for step 9**

**Timing: 10 min for step 10**

**Timing: 5 min for step 11**

**Timing: this depends on the amount of primers that are added to the primer pool for step 13.**

**CRITICAL:** Make sure to use tubes, pipette tips, reagents, etc. that are suitable for PCR experiments and are therefore free of DNA and free of DNase/RNase.

8. Aliquotting the Single Cell-to-CT qRT-PCR Kit (Thermo Fisher Scientific)
  **CRITICAL:** Prepare single use aliquots of the Single Cell-to-CT qRT-PCR Kit (ThermoFisher Scientific) in e.g. 0.5 mL tubes in order to prevent thaw-freeze cycles and possible contaminations of the reagents.
  a. Thaw all the reagents on ice and freeze them right after aliquotting. The Single Cell Lysis Solution is stored at 4°C.
  b. The Single Cell-to-CT qRT-PCR Kit has the same shelf life for all reagents in the kit. The shelf life is usually around 9 months upon receipt.
  c. It might be possible to reduce the volume of the aliquots and therefore increase the number of tubes and runs possible.
  ***Note:*** The Single Cell PreAmp is difficult to pipette due to its viscosity. Pay attention to pipette the respective amount.

**Table:** 

Name	Volume per tube [μL]	Needed per run [μL]	Per aliquot (with overage) [μL]	Overage Amount [μL]	# Of tubes prepared
Single Cell Stop Solution	50	1.94	2.5	0.56	18
Single Cell Pre-Amp Mix	265	12.0	14.0	2.0	18
Single Cell SuperScript RT	75	3.62	4.0	0.38	18
Single Cell VILO RT Mix	150	5.84	7.0	1.16	18
Single Cell Dnase I	50	1.4	2.5	1.1	18
Single Cell Lysis Solution	500	12.75	15.0	2.25	18

9. Aliquotting C1 Dilution Reagent (Fluidigm) and Cell Wash Buffer (Fluidigm)

Aliquot the C1 Dilution Reagent as 2.8 mL aliquots (or 2× 1.4 mL when using 1.5 mL tubes) and the Cell Wash Buffer as 100 μL aliquots to decrease thawing time and prevent contaminations between the runs.

10. Aliquot ROX Reference Dye (Thermo Fisher Scientific)

Aliquot the ROX Reference Dye into single use aliquots of 8 μL. The ROX Reference Dye is light sensitive. Store the aliquots at −20°C and in the dark.

11. Prepare FACS Buffer PBS with 2% FBS.

12. Order individual primers and resuspend individual primers if needed

If necessary, order any Delta Gene primers that are needed for the pre-amplification individually through a different primer production company and resuspend the primers at 100 μM.

For example for pre-amplification of the SNP region rs3853839 the reverse Delta Gene primer needs to be ordered individually, as Fluidigm provides the Delta Gene primers as equimolar pooled of reverse and forward primers. Refer to Figure 1 and the section *Before You Begin* for clarification.

Up to 200 primer pairs can be added to the primer pool. To be able to indeed add 200 primer pairs to the primer pool resuspend the primer pair that is needed for pre-amplification of the SNP region as an equimolar pool of forward and reverse primer of 100 μM.

13. Pipette the primer pool
  a. Thaw the primers on ice.
  b. Thoroughly vortex and spin the primers and then combine 1 µL of all primers into a fresh tube.
  c. Fill it up to 200 μL with the C1 Dilution Reagent (Fluidigm).
  d. Aliquot the primer pool for single use aliquots of 8 μL to avoid contaminations between the runs and to avoid thaw-freeze cycles.

***Note:*** You can use these primer pool aliquots for up to 1 year.

For targets that are only included for gene expression analysis (e.g., the reference gene B2M) add the Delta gene primers to the primer pool.

For the SNP regions combine the outer most primers of the SNP Type / Delta Gene primers in order to generate an amplicon that can subsequently be analyzed by the SNP Type and Delta Gene primers separately. Refer to Figure 1 and the section *Before You Begin* for clarification.

Also refer to p. 15 of the *Using C1 to Capture Cells from Cell Culture and Perform Preamplification Using Delta Gene Assays* (PN100-4904 L1) that can be accessed here:

https://www.fluidigm.com/binaries/content/documents/fluidigm/resources/c1-delta%E2%80%90gene-pr-100%E2%80%904904/c1-delta%E2%80%90gene-pr-100%E2%80%904904/fluidigm%3Afile

## Key resources table

**Table:** 

REAGENT or RESOURCE	SOURCE	IDENTIFIER
**Antibodies**		

CD11c-PE/Cy7 (Bu15)	BioLegend	Cat#: 337216 RRID: AB_2129790
CD123-FITC (6H6)	BioLegend	Cat#: 306014 RRID: AB_2124259
CD14-APC/Cy7 (HCD14)	BioLegend	Cat#: 325620 RRID: AB_830693
CD19-BUV737 (SJ25C1)	BD	Cat#: 564303 RRID: AB_2716867
CD3-BUV737 (UCHT1)	BD	Cat#: 564307 RRID: AB_2744390
CD56-BUV395 (NCAM16.2)	BD	Cat#: 563554 RRID: AB_2687886
HLA-DR-BV605 (L243)	BioLegend	Cat#: 307640 RRID: AB_2561913

**Biological samples**		

Human blood	Healthy individuals from the University Medical Center Hamburg-Eppendorf	Cat#: N/A

**Chemicals, peptides, and recombinant proteins**		

DNA Suspension Buffer, pH 8.0, DNase/RNase Tested, PCR Grade	Teknova	Cat#: T0221
Sso Fast EvaGreen Supermix with Low ROX	Bio-Rad	Cat#: 1725210
Fast Probe Master Mix	Biotium	Cat#: 31005
Aqua ad iniectabilia (DNA-free water)	Braun	Cat#: 235 1744
ACK Lysing Buffer	Lonza	Cat#: 10-548E
Biocoll-Trennlösung	Biochrom	Cat#: L6115
Dulbecco’s Phosphate Buffered Saline (PBS)	Sigma-Aldrich	Cat#: D8537
Dymethyl sulfoxide (DMSO)	Sigma-Aldrich	Cat#: D5879-100ML
Fetal bovine serum (FBS) superior	Biochrom	Cat#: S0615
ROX Reference Dye	Thermo Fisher Scientific	Cat#: 12223012
RPMI-1640 Medium	Life Technologies	Cat#: 21875091

**Critical commercial assays**		

C1 Single-Cell Reagent Kit for Preamp	Fluidigm	Cat#: 100-5319
DNeasy Blood & Tissue Kit (250)	QIAGEN GmbH	Cat#: 69506
GE 96.96 Dynamic Array™ DNA Binding Dye Sample & Assay Loading Reagent Kit with Control Line Fluid	Fluidigm	Cat#: 100-3415
QIAGEN Multiplex PCR Kit	QIAGEN	Cat#: 206143
Plasmacytoid Dendritic Cell Isolation Kit II, human	Miltenyi Biotec	Cat#: 130-097-415
Single Cell-to-CT qRT-PCR Kit	Thermo Fisher Scientific	Cat#: 4458237
SNP Type™ 192.24 Genotyping Reagent Kit with Control Line Fluid	Fluidigm	Cat#: 100-4136
Zombie Aqua™ Fixable Viability Kit	BioLegend	Cat#: 423102

**Oligonucleotides**		

*CYBB -* pre-amplification – forward primer AAGGAAATTTTCCAGATCATTAGGACA	This paper	N/A
*CYBB -* pre-amplification – reverse primer CCCAGTTACCCTGCTGTATTAGTA	This paper	N/A
*CYBB –* quantitative PCR – forward primer GAGAGTGTCTCAACACTTATTAGTGAC	This paper	N/A
*CYBB –* quantitative PCR – reverse primer CCCAGTTACCCTGCTGTATTAGTA	This paper	N/A
*CYBB -* SNP typing (rs5964151) - forward primers ACATGTTGAGAGTGTCTCAACACTTAT ACATGTTGAGAGTGTCTCAACACTTAG	This paper	N/A
*CYBB -* SNP typing (rs5964151) - reverse primer GGAGTATGCTCAGATGTCAATACTGTCA	This paper	N/A
*B2M -* pre-amplification + quantitative PCR – forward primer TTAGCTGTGCTCGCGCTAC	This paper	N/A
*B2M -* pre-amplification + quantitative PCR – reverse primer CTCTGCTGGATGACGTGAGTAA	This paper	N/A
*IRF2BP2 -* pre-amplification + quantitative PCR – forward primer GGCCCTTCGAGAGCAAGTTTAA	This paper	N/A
*IRF2BP2 -* pre-amplification + quantitative PCR – reverse primer TGGTTCTGGAGAGGGCTTCC	This paper	N/A
*TLR7 -* pre-amplification – forward primer TGGGCACCACACAGGT	This paper	N/A
*TLR7 -* pre-amplification – reverse primer CTGTTTCCCTATGGAACCCAGAA	This paper	N/A
*TLR7 -* quantitative PCR – forward primer ACAGGTGGTTGCTGCTTCA	This paper	N/A
*TLR7 -* quantitative PCR – reverse primer CTGTTTCCCTATGGAACCCAGAA	This paper	N/A
*TLR7 -* SNP typing (rs3853839) – forward primers CTTCAGTGCTTCCTGCTCTTTTTC CTTCAGTGCTTCCTGCTCTTTTTG	This paper	N/A
*TLR7 -* SNP typing (rs3853839) – reverse primer CTATGGAACCCAGAAGCAGGC	This paper	N/A

**Software and algorithms**		

CLC Main Workbench, version 7.9.1	QIAGEN Aarhus A/S	https://digitalinsights.qiagen.com/
FlowJo10	FlowJo LLC	http://www.flowjo.com
Fluidigm Real-Time PCR Analysis, version 4.3.1	Fluidigm	https://www.fluidigm.com/
Fluidigm SNP Genotyping Analysis; version 4.3.2	Fluidigm	https://www.fluidigm.com/
GraphPad Prism 9	GraphPad Software, LLC	https://www.graphpad.com/
Microsoft PowerPoint 2016	Microsoft	https://www.microsoft.com

**Other**		

192.24 Dynamic Array™ IFC for SNP Genotyping	Fluidigm	BMK-M-192.24GT
48.48 Dynamic Array™ IFC for Genotyping	Fluidigm	BMK-M-48.48GT
96.96 Dynamic Array™ IFC for Gene Expression	Fluidigm	BMK-M-96.96
C1™ Single-Cell Preamp IFC, 5–10 μm	Fluidigm	100-5757
qPCR foil	SARSTEDT	95.1994
PCR plate half skirt, 96 well	SARSTEDT	72.1979.102
Sterican single-use cannula, blunt (Ø 0.8 × 22 mm)	Braun	918 0109
8-Channel pipette (0.5 μL–10 μL)	Rainin	17013802
8-Channel pipette (5 μL–50 μL)	Rainin	17013804
C1	Fluidigm	N/A
Biomark HD	Fluidigm	N/A
Juno	Fluidigm	N/A
Fluorescence-activated cell sorter (FACS), e.g., FACSAria Fusion	BD	N/A
Microscope with 20× and 40× magnification	N/A	N/A
NanoDrop	N/A	N/A

## Step-by-step method details

**CRITICAL:** Make sure to use tubes, pipette tips, reagents, etc. that are suitable for PCR experiments and are therefore free of DNA and free of DNase/RNase.

### Using the C1 to generate pre-amplified cDNA of individual captured cells

**Timing: this is variable depending on the processing time of cells for step 1**

**Timing: 20 min for step 2**

**Timing: 15 min (for the C1 Single-Cell Preamp IFC, 5–10 μm) for step 3**

**Timing: depends on the percentage of target cell in cell population for step 4**

**Timing: 30–40 min for step 5**

**Timing: 20 min, the Preamp program runs for 5–6 h for step 6**

**Timing: 60 min for step 8**

**Timing: variable, depending on how many donors and conditions will be pooled for step 9**

The following steps were adapted from the protocol *Using C1 to Capture Cells from Cell Culture and Perform Preamplification Using Delta Gene Assays* (PN 100–4904 L1) :

https://www.fluidigm.com/binaries/content/documents/fluidigm/resources/c1-delta%E2%80%90gene-pr-100%E2%80%904904/c1-delta%E2%80%90gene-pr-100%E2%80%904904/fluidigm%3Afile

There is the possibility to include RNA spikes as an additional control. Refer to the protocol for more information. Fluidigm reagents should be thawed to 20°C–24°C, vortexed thoroughly and spun down prior to usage. Frozen, non-Fluidigm reagents should be thawed and stored on ice, vortexed thoroughly and spun down prior to usage. Cell Wash Buffer (Fluidigm) and the Single Cell Lysis Solution (Thermo Fisher Scientific) are stored at 4°C and should be kept on ice for the duration of the experiment. Keep non-Fluidigm reagents and mixes containing non-Fluidigm reagents dark and on ice. The Fluidigm reagents used in this section are from the C1 Single-Cell Reagent Kit for Preamp (Fluidigm, Cat#: 100-5319).

1. Preparation and staining of cells
  The length of the steps depend on whether you use freshly isolated or frozen cells and whether you would like to investigate unstimulated or stimulated cells. When using cells from a cell line or an organoid culture, it could also be possible to omit the sorting step. In that case determine your cell number and mix your cells with the Suspension Reagent (Fluidigm). For obtaining pDCs from PBMCs it is possible to perform a pre-enrichment before sorting, but that is only necessary if they will be stimulated to exclude the influence from other cell types. Otherwise it is possible to sort from bulk PBMCs, since only 2000–4000 individual cells are needed. Sorting from PBMCs decreases the processing time.
  a. Resuspend 12.5×10^6^ PBMCs (to ensure to be able to sort 2000–4000 pDCs) with 31.25 μL of surface antibodies and viability stain in 250 μL PBS for 20 min at 20°C–24°C in the dark. The volume of the antibody and staining volume were scaled up accordingly (×2.5) from the values in Table 1.

**Table 1:** Antibody mix for staining of PBMCs (for up to 5**×**10^6^ PBMCs in 100 μL PBS, scale up accordingly, e.g., 10**×**10^6^ PBMCs are stained with 25 μL antibody mix in 200 μL PBS)

Reagent	Dilution	Amount [µL]
Zombie Aqua™ Fixable Viability Kit (pre-diluted 1:10 in PBS)	1:1000	1.0
CD14-APC/Cy7	1:100	1.0
CD3-BUV737	1:100	1.0
CD19-BUV737	1:100	1.0
CD123-FITC	1:67	1.5
CD11c-PE/Cy7	1:67	1.5
CD56-BUV395	1:40	2.5
HLA-DR-BV605	1:33	3.0
Total Volume	N/A	12.5
  b. After 20 min, add 3 mL PBS and centrifuge (7 min / 500 g).
  c. Discard the supernatant.

**CRITICAL:** keep the stained cells dark and on ice.

***Note:*** It is recommended to run the sample at your cell sorter, before starting with the next steps.

**Figure 2:**
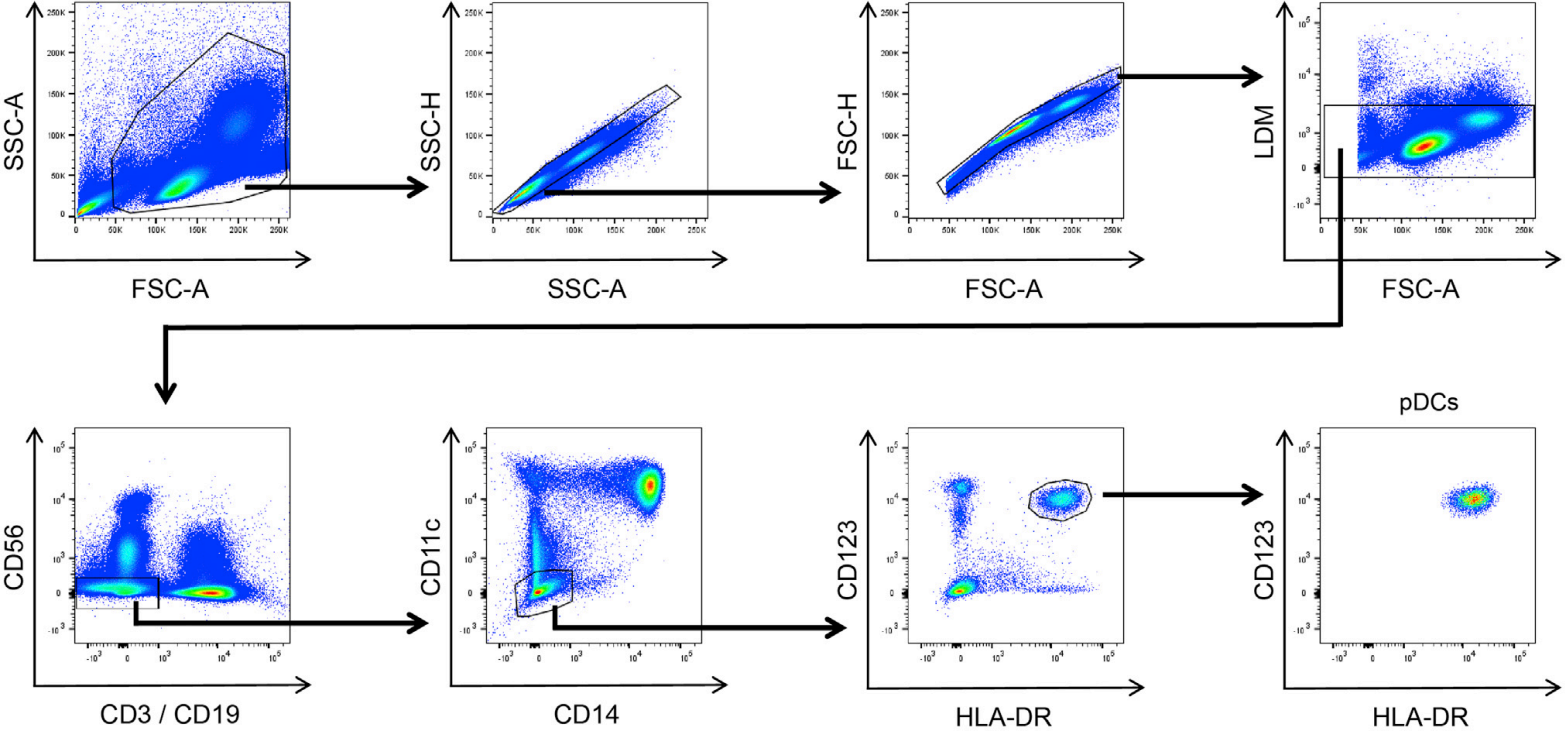
Gating strategy to sort human pDCs from human PBMCs pDCs were defined as single, live and CD3-CD19-CD56-CD11c-CD14-CD123+HLA-DR+. LDM: live-dead marker. The gating strategy was adapted with permission from (Hagen et al., 2020).

pDCs were defined as single and live CD3-CD19-CD56-CD11c-CD14- and HLA-DR+ and CD123+. For the gating strategy refer to Figure 2.

2. Preparation of the mixes for cell lysis, reverse transcription and pre-amplification
  These are the different mixes for the cell lysis, reverse transcription and pre-amplification of the regions of interest.
  **CRITICAL:** Pipette the mixes in a pre-PCR area.
  a. Prepare the Reverse Transcription Final Mix and the Preamp Final Mix according to company protocol on p. 16–17 (the pooled primers were prepared before).
    ***Note:*** Pay attention when pipetting the Single Cell PreAmp Mix (Thermo Fisher Scientific) as it is difficult to pipette due to the viscosity of the liquid.
  b. The Adapted Lysis final mix is prepared as follows:
    i. Prepare a Lysis/DNase solution:
    1.4 μL of DNase I solution
    12.6 μL of Single-Cell lysis solution
    ii. Adapted Lysis Final Mix:
    12.75 μL Lysis/DNase solution
    4.35 μL C1 Lysis Plus Reagent
    0.9 μL C1 DNA Dilution Reagent
  c. Store the prepared mixes dark and on ice until usage.

3. Priming of the C1 IFC
  a. Priming of the C1 IFC is done according to the protocol on page 19.
  b. After priming the C1 IFC should be loaded with the cells within 1 h.
  c. For pDCs and cells of a comparable size, use the C1 Single-Cell Preamp IFC, 5–10 μm.

4. Sorting of the cells into the C1 IFC
  For calculations and an extended protocol for sorting of the cells into the C1 IFC, refer to the Fluidigm guide *Cell Sorting Directly to the C1 IFC* (PN 101–5826 A2) and the excel sheet *Reagent Calculations_Sorting Into C1 IFC*:
  https://www.fluidigm.com/articles/cell-sorting-directly-to-the-c1-ifc
  The cell number/concentration and the buoyancy of the cells is very critical in order to achieve a high capture rate of the cells within the C1 IFC. Sorting of the cells gives the best control over purity and cell number and with a small cell population it is recommended to sort directly into the C1 IFC.
  It is also possible to sort the cells into a different tube (e.g., PCR tube) and then transfer them afterward into the C1 IFC.
  Use a 70 μm nozzle to ensure small sorting volume (1000 cells will be in roughly 1 μL volume), and keep the sorting speed at 1.0. Make sure to select single cell sort. We sorted with a FACSAria Fusion from BD, please adapt the settings according to your cell sorter. The cells were sorted at 4°C. For pDCs we aimed at cell concentration of 420 pDCs / μL (3360 pDCs in 8 μL), with adding 1 μL Suspension Reagent (Fluidigm) for 3 μL of cell suspension (2 μL of Suspension Reagent in a total volume of 8 μL). Roughly 5 μL of cell suspension will be loaded on the C1 which are under these conditions around 2100 pDCs total.
  a. If you have a limited number of cells, it can be helpful to set up the sorting gates with a reference sample.
  b. For the gating strategy for human pDCs see Figure 2.
  c. Remove the C1 Blocking Reagent from the cell inlet and the cell outlet of the C1 IFC (do this shortly before the sort).
  d. Add 1 μL of FACS Buffer to the cell inlet.
  e. Sort 3360 pDCs into the cell inlet of the C1 IFC (which will be equivalent to roughly 3.4 μL of volume with the FACSAria Fusion and the settings explained above).
  f. If you have more cells you can sort them into a PCR Tube containing 1 μL of FACS buffer and then use those later for the Tube Control (Positive Control).
  g. Add 1.6 μL of FACS Buffer.
  h. Thoroughly vortex and spin the Suspension Reagent before adding 2.0 μL to the cell inlet.
  i. Mix the cell suspension by pipetting up and down 15 to 20 times. Make sure to avoid the creation of bubbles.
  j. Make sure that you have more than 6 μL of volume in the cell inlet (during the loading step 5 μL of the cell suspension will be taken up into the IFC).
  k. If you have more cells it is possible to upscale the volume, but make sure to keep the cell concentration around 420 pDCs / μL and the cell suspension to Suspension Reagent ratio constant (1 μL Suspension Reagent for 3 μL of cell suspension).
  l. Let the cells adjust to the Suspension Reagent for 10 min.
  m. Visualize the cell inlet under a microscope to make sure that you see your cells of interest in the cell inlet. The cells should be distributed throughout the different levels.
  n. Following the 10 min incubation time place the IFC into the C1 and select the STA: Cell Load program.

5. Imaging of the cells
  It is helpful to use the Excel file *C1 Sample Attribution* from Fluidigm which gives an automatic overview of the individual capture sites in a 96-well plate Layout.
  a. Visualize each Capture Site under a microscope.
  b. It is important that there is only one cell (without any cell debris) in the entire capture site.
  c. Mark capture sites that are completely empty as those can be used as an additional negative control.
  d. Indicate for each capture site how many cells you observe and whether you can see cell debris. Only capture sites with 1 individual cell will be used going forward, but the information about all capture sites can be used to judge whether the cell concentration should be increased or decreased if the capture rate is not satisfying.

6. Run Lysis, Reverse Transcription and Preamplification
  a. After visualization, add the Harvest Reagent, (adapted) Lysis Final Mix, RT Final Mix and Preamp Final Mix to the IFC according to p. 24.
    **CRITICAL:** Make sure that there are no bubbles in any of the liquids. If you can see bubbles make sure to destroy them, e.g., with a pipette tip.
  b. Place the IFC into the C1 and start the Preamp program. The program takes 5–6 h (depending on the IFC).

**Pause point:** The harvest can be scheduled for up to roughly 21 h - 22 h after the start of the program.

7. Negative and positive Control
  a. Use 1 μL of cells that were sorted into a PCR tube for the positive control.
  b. Use 1 μL of Cell Wash Buffer for the negative control.
  c. Follow the steps outlined on p. 32–34 in the protocol.
8. Harvest of amplified products
  a. Eject the C1 IFC from the C1 and transfer it into a post PCR environment.
  b. Aliquot 25 μL of C1 DNA Dilution Reagent into each well of a 96 well plate by adding first 190 μL and then 120 μL of C1 DNA Dilution Reagent in an 8er PCR stripe and transferring 25 μL with a 8-channel pipette.
  c. Harvest the amplicons according to p. 26–30 of the C1 Protocol.
  d. When harvesting the amplicons set the 8-channel pipette to 3.6 μL.
  e. Seal the plate with a qPCR foil.
  f. After the harvest vortex the plate thoroughly and then spin it down (1000 g / 1 min).
  g. Store the harvested amplicons (“diluted harvest plate”) at −20°C.

**Pause point:** After freezing of the amplified cDNAs, the analysis of the cDNA can be done at a later time. It is recommended to pool the different cDNAs; see next step 9. Pooling of cDNA.

9. Pooling of cDNA

When investigating multiple donors or conditions it is recommended to transfer the harvested amplicons of the different donors / conditions equally over multiple 96 well plates in order to reduce a possible batch effect of the gene expression analysis. While pooling of the cDNA only include wells from capture sites that included one single cell. This reduces the amount of gene expression and SNP Typing IFCs that will need to be run. Include one water control on every plate. Include your positive and negative control for every C1 run. It is possible to include empty capture sites in addition to the negative control as it is possible that a contamination occurred during processing of the tube control (refer to *Problem 5*).

**CRITICAL:** When investigating the escape from XCI of a new gene, always perform the SNP typing of the mRNA with a female that is homozygous for the respective SNP or with a male individual (for genes lying outside of the pseudoautosomal region of the X chromosome) to verify specificity of the mRNA SNP typing. For the SNP typing run of the mRNA it is recommended to include gDNAs with a known genotype (without STA performed).

### Gene expression of pre-amplified samples with the 96.96 IFC using Delta Gene Assays

**Timing: 5–6 h**

**Timing: 1 h (once everything is thawn) for step 10**

**Timing: 1 h (once everything is thawn) for step 11**

**Timing: 10 min + the protocol “Prime 96.96 GE” takes roughly 21 min for step 12**

**Timing: 20–30 min for step 13**

**Timing: 20–30 min for step 14**

**Timing: The protocol “Load Mix 96.96 GE” takes roughly 92 min for step 15**

**Timing: 10 min + this program runs for roughly 75 min for step 16**

There are different formats for the gene expression analysis. We used the 96.96 IFC for gene expression (analyzing the expression of 96 mRNAs in 96 individual cells). For the gene expression analysis only the Delta Gene primers are employed (see Figure 1B).

The following steps were adapted from the Quick Reference Guide *Gene Expression with the 96.96 IFC Using Delta Gene Assays on Preamplified Samples* (PN 100–9792 B1):

https://www.fluidigm.com/binaries/content/documents/fluidigm/resources/96.96-ge-delta-gene-qr-100-9792/96.96-ge-delta-gene-qr-100-9792/fluidigm%3Afile

Also refer to the *Biomark HD Data Collection* user guide (100–2451 Rev 14):

https://www.fluidigm.com/binaries/content/documents/fluidigm/resources/biomark-hd-data-collection-ug-100-2451/biomark-hd-data-collection-ug-100-2451/fluidigm%3Afile

The steps are written for the Instruments Juno and Biomark HD. When using different instruments, please refer to the guides listed above.

Fluidigm reagents should be thawed to 20°C–24°C, vortexed thoroughly and spun down prior to sample and assay mix preparation. Thaw and store the primer plates on ice and in the dark. Thoroughly vortex the primer plates and spin them down (1000 g / 1 min), prior to use. Frozen, non-Fluidigm reagents should be thawed and stored on ice, vortexed thoroughly and spun down prior to sample and assay mix preparation. Keep non-Fluidigm reagents and mixes containing non-Fluidigm reagents dark and on ice. The Fluidigm reagents used in this section are from the GE 96.96 Dynamic Array™ DNA Binding Dye Sample & Assay Loading Reagent Kit with Control Line Fluid (Fluidigm, Cat#: 100–3415).

Always include a water sample.

**CRITICAL:** Vortex thoroughly and centrifuge all assay and sample solutions before pipetting into IFC inlets. Avoid bubbles.

***Note:*** Do not refreeze the Sso Fast EvaGreen Supermix. It is possible to use it for up to 6 month, when it is stored at 2°C–4°C and in the dark (thus it is also possible to thaw the Sso Fast EvaGreen Supermix the day before at 2°C–4°C).

10. Preparation of the Assay (Primer) plate (on a 96 well plate)
  ***Note:*** If multiple Gene expression runs are planned prepare an Assay plate that can be used for multiple runs. This saves time and reduces potential variability between the runs.
  The Assay plate consists of the Delta Gene primers, the 2× Assay Loading Reagent and the DNA Suspension Buffer (Teknova, Cat#: T0221).
  ***Note:*** The Assay plate can be used for up to three weeks at −20°C or 10 thaw-freeze cycles.
  a. Take 2× Assay Loading Reagent and DNA Suspension Buffer and vortex both thoroughly and spin them down.
  b. In a new tube combine for the Assay Mix of one plate (we increased the volume of one plate (×1,5), to increase the volume of primer that needs to be transferred from 0.3 μL to 0.45 μL):

**Table:** 

	Vol. per inlet	Vol. per inlet with overage	Vol. For 96.96 IFC
2× Assay Loading Reagent (Fluidigm)	2.5 µL	3.0 µL	450 μL
DNA Suspension Buffer (Teknova, Cat#: T0221)	2.25 µL	2.7 µL	405 μL
Total Volume			855 μL
    Or for multiple plates (the numbers are for 2 plates – scale up accordingly):

**Table:** 

	Vol. per inlet	Vol. per inlet with overage	Vol. For 96.96 IFC
2× Assay Loading Reagent (Fluidigm)	2.5 µL	3.0 µL	600 μL
DNA Suspension Buffer (Teknova, Cat#: T0221)	2.25 µL	2.7 µL	540 μL
Total Volume			1140 μL
  c. Vortex the Assay Mix and spin it down and then pipette 106 μL (2× 53 μL – use the 100 μL pipette) from the Assay Mix into each tube of an 8 PCR Stripe.
  d. Take a new 96 Well PCR Plate (Assay Plate) and aliquot 8.55 μL of the Assay Mix from the 8 PCR Stripe into each well of the Assay Plate by using the 8-channel pipette.
  e. Make sure to stop at the first stop of the pipette and thus avoid the creation of bubbles.
  f. Transfer 0.45 μL (0.6 μL for two plates – scale up accordingly) from the Primer Plate to the Assay Plate. Make sure to keep the layout of the Primer Plate.
  g. Seal the plate with a qPCR foil.
  h. Vortex the plate thoroughly (make sure to vortex the entire plate) and spin it down (1000 g, 1 min).
  i. Store the plate on ice and in the dark.

**Pause point:** As the Assay plate can be used for up to 3 weeks when stored at −20°C, the Assay plate can be prepared in advance.

11. Preparation of the Sample (cDNA) plate (on a 96 Well PCR Plate)
  ***Note:*** Always prepare the Sample plate fresh.
  a. Take SsoFast EvaGreen (very light sensitive + tends to foam a lot) and 20× DNA Binding Dye Sample Loading Reagent and vortex both thoroughly for 20 s.
  b. In a new tube combine for the Sample Mix:

**Table:** 

	Vol. per inlet	Vol. per inlet with overage	Vol. For 96.96 IFC
Sso Fast EvaGreen Supermix (Biorad, Cat#: 1725210)	2.5 µL	3.0 µL	360 μL
20× DNA Binding Dye Sample Loading Reagent (Fluidigm)	0.25 µL	0.3 µL	36 μL
Total Volume			396 μL
  c. Vortex the pipetted Sample Mix and centrifuge.
  d. Pipette 45 μL from the Sample Mix into each tube of a 8 PCR Stripe.
  e. Take a new 96 Well PCR Plate and aliquot 3.3 μL of the Sample Mix from the 8 PCR Stripe into each well of a 96 Well PCR Plate (Sample Plate) by using the 8-channel pipette.
  f. Transfer 2.7 μL of cDNA from the pooled cDNA plate (see *Pooling of cDNA*) or from the diluted harvest plate 96 Well PCR Plate (see *Harvest of amplified products*) to the Sample Mix. Make sure to keep the layout of the pooled cDNA.
  g. Seal the Sample Plate with a qPCR foil.
  h. Vortex the plate thoroughly (make sure to vortex the entire plate) and spin it down (1000 g, 1 min).
  i. Store it on ice and in the dark.

12. Prime the 96.96 GE IFC
  The following steps were adapted from *Control Line Fluid Loading Procedure* (PN 68000132 r07):
  https://www.fluidigm.com/binaries/content/documents/fluidigm/resources/control-line-fluid-loading-procedure-68000132-qr/control-line-fluid-loading-procedure-68000132-qr/fluidigm%3Afile
  **CRITICAL:** The control line fluid inhibits the read-out of the data, so make sure, that it does not drop on the IFC. If the control line fluid was spilled on the IFC, try to carefully remove it by using 70% Ethanol.
  **CRITICAL:** Wear safety goggles while applying the control line fluid.
  a. Before opening the 96.96 IFC, evacuate carefully the air out of the syringe.
  b. Open the package of the 96.96 IFC.
  c. Using a 100 μL pipette tip make sure that both valves open easily.
  d. Take the 96.96 IFC, put it into a horizontal orientation and tilt it 45°.
  e. Start with the valve that is now situated on the bottom of the plate.
  f. Using the syringe to press down the valve.
  g. Visually confirm that the valve is open.
  h. Inject the control line fluid into the IFC by slowly pushing down the plunger of the syringe.
  i. After completely emptying the syringe, wait a few seconds before removing the syringe.
  j. It is normal that there is some liquid remaining in the syringe.
  k. Verify that the O-ring returns to its normal position after syringe removal.
  l. Make sure that no liquid was spilled.
  m. Turn the plate 180°.
  n. Fill up the other valve according to steps f) – l).
  o. Put the IFC into the Juno and run the following script: Prime 96.96 GE.

You have now 1 h time to pipette Assays and Samples on the 96.96 IFC and start the load protocol on the Juno.

**Figure 3:**
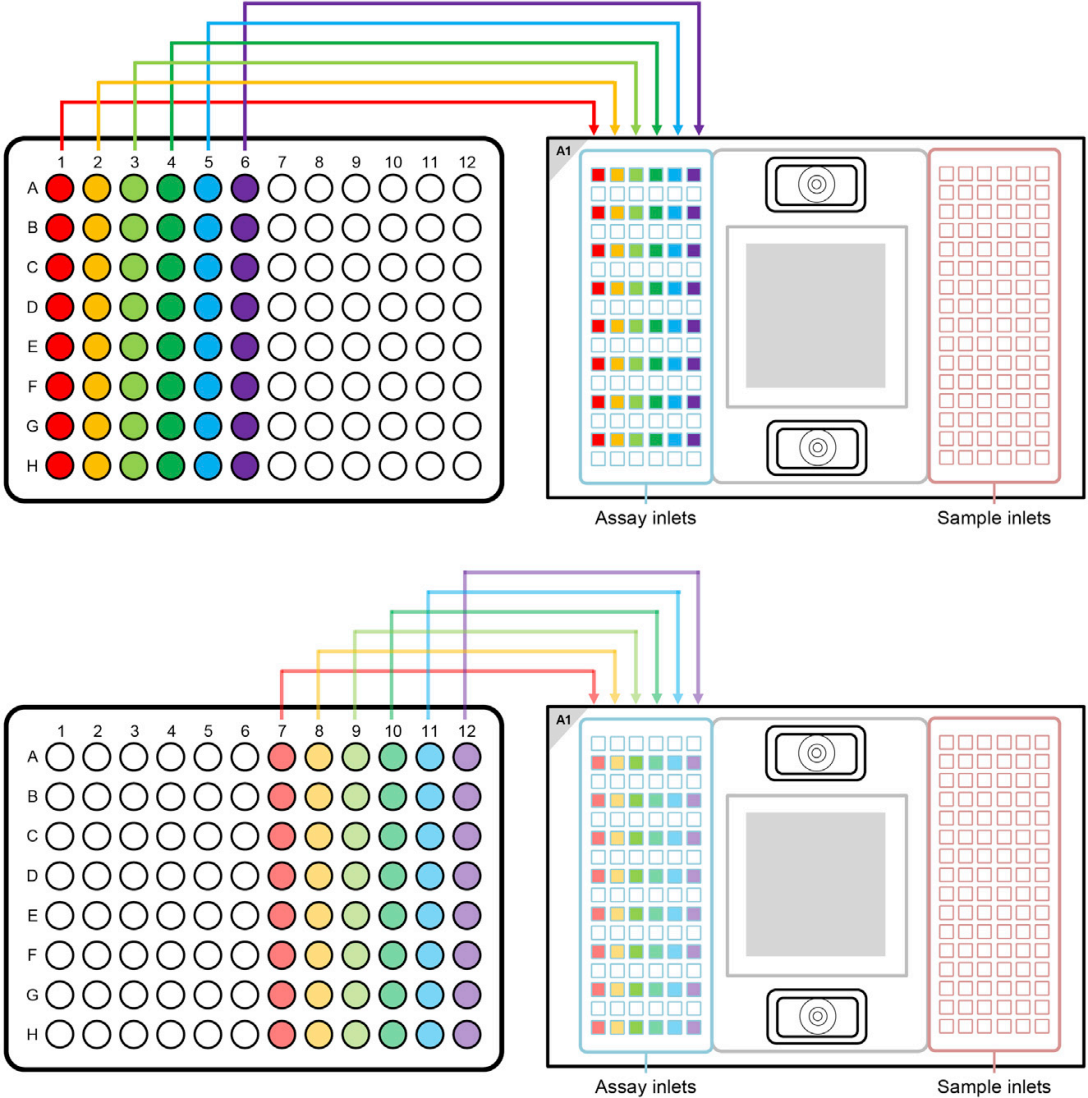
Pipetting scheme for transferring the assays / primers on a 96.96 gene expression IFC The Figure was recreated based on figures and information from the Quick Reference Guide *Gene Expression with the 96.96 IFC Using Delta Gene Assays on Preamplified Samples* (PN 100-9792 B1): https://www.fluidigm.com/binaries/content/documents/fluidigm/resources/96.96-ge-delta-gene-qr-100-9792/96.96-ge-delta-gene-qr-100-9792/fluidigm%3Afile and the protocol *Using C1 to Capture Cells from Cell Culture and Perform Preamplification Using Delta Gene Assays* (PN 100-4904 L1): https://www.fluidigm.com/binaries/content/documents/fluidigm/resources/c1-delta%E2%80%90gene-pr-100%E2%80%904904/c1-delta%E2%80%90gene-pr-100%E2%80%904904/fluidigm%3Afile
  d. Do not leave any wells empty.
  e. To every unused assay inlet, add either 3.0 μL assay loading reagent and 3.0 μL DNA-free water or 5.7 μL Assay Mix and 0.3 μL of DNA-free water.

13. Transferring the Assays (primers) on the 96.96 IFC
  **CRITICAL:** Vortex the Assay Plate thoroughly and spin down (1 min / 1000 g) before loading them on the 96.96 IFC.
  a. Always halt at the first stop of the pipette when pipetting. Try to avoid bubbles.
  b. Pipette 5 μL with an 8-channel pipette from each assay from the Assay Plate on the 96.96 IFC.
  c. Pipette the Assays on the left side of the IFC (see Figure 3).

**CRITICAL:** After pipetting of the Assays on the 96.96 IFC, check for bubbles and destroy all of them using a pipette tip or hollow needle / cannula. Use a fresh pipette tip / cannula for every well.

**Figure 4:**
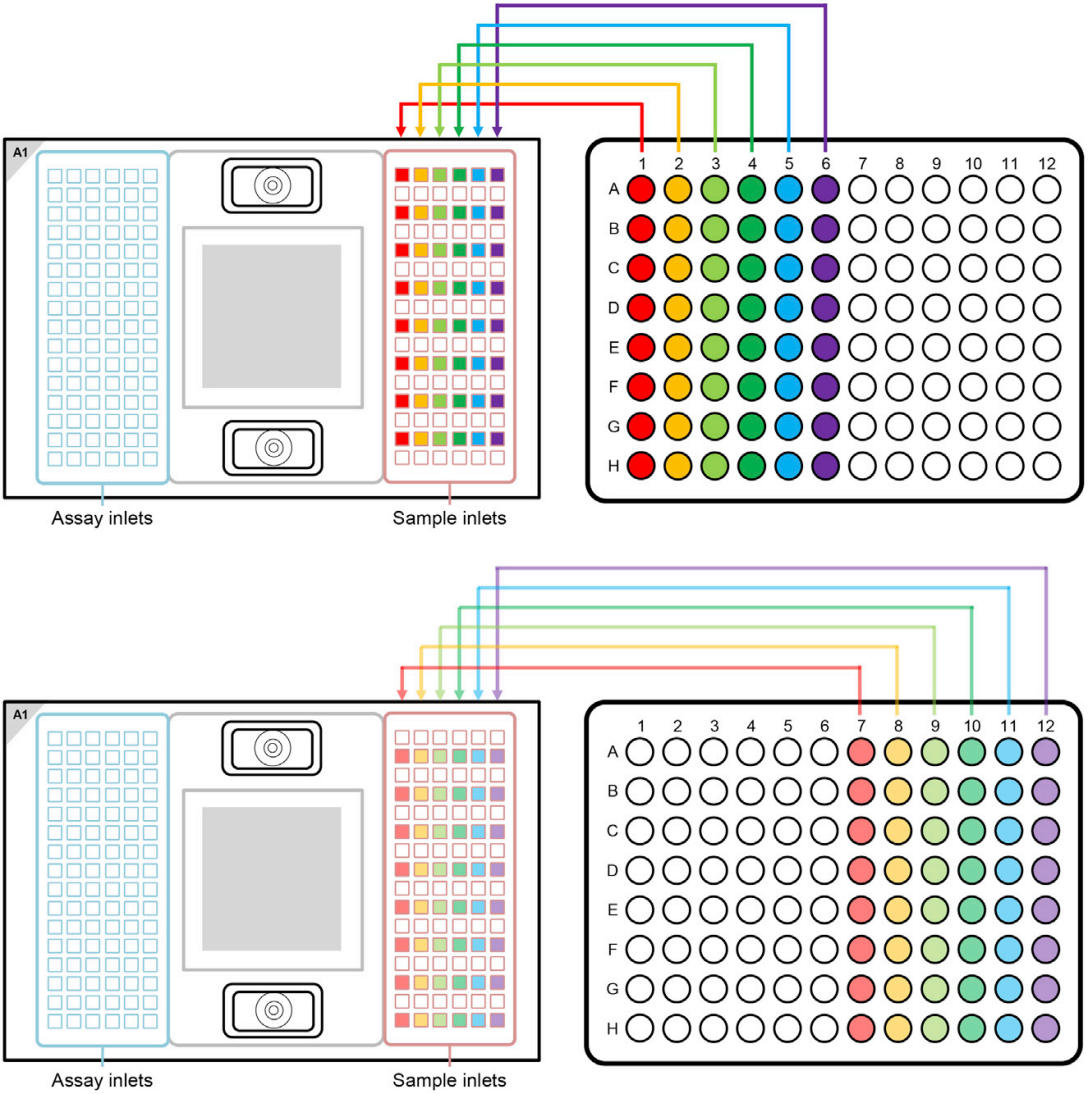
Pipetting scheme for transferring the samples / cDNA to a 96.96 gene expression IFC The Figure was recreated based on figures and information from the Quick Reference Guide *Gene Expression with the 96.96 IFC Using Delta Gene Assays on Preamplified Samples* (PN 100-9792 B1): https://www.fluidigm.com/binaries/content/documents/fluidigm/resources/96.96-ge-delta-gene-qr-100-9792/96.96-ge-delta-gene-qr-100-9792/fluidigm%3Afile and the protocol *Using C1 to Capture Cells from Cell Culture and Perform Preamplification Using Delta Gene Assays* (PN 100-4904 L1): https://www.fluidigm.com/binaries/content/documents/fluidigm/resources/c1-delta%E2%80%90gene-pr-100%E2%80%904904/c1-delta%E2%80%90gene-pr-100%E2%80%904904/fluidigm%3Afile
  d. To every unused sample inlet, add 3.3 μL of sample mix and 2.7 μL of DNA-free water.

14. Transferring the samples (cDNA) on the 96.96 IFC
  **CRITICAL:** Reduce light exposure when pipetting the samples, since EvaGreen is light sensitive.
  ***Note:*** The samples are more prone to produce bubbles, be extra careful when pipetting.
  **CRITICAL:** Vortex the Sample plate thoroughly and spin down (1 min / 1000 g) before loading them on the 96.96 IFC.
  a. Always halt at the first stop of the pipette when pipetting. Try to avoid bubbles.
  b. Pipette 5 μL with an 8-channel pipette from each sample from the Sample plate to the 96.96 IFC.
  c. Pipette the samples on the right side of the IFC (see Figure 4).

**CRITICAL:** After pipetting of the samples on the 96.96 IFC, check for bubbles and destroy all of them using a pipette tip or hollow needle / cannula. Use a fresh pipette tip / cannula for every well.

15. Load Mix of the 96.96 GE IFC

***Note:*** Start the IFC run within 1 h of adding the assays and samples on the IFC.

Depending on the layout of the IFC (96.96 or 192.24) the Juno needs a different interface plate. Refer to p. 27ff of the *Juno System* user guide (PN 100–7070 r12) on how to change the interface plate:

https://www.fluidigm.com/binaries/content/documents/fluidigm/resources/juno-user-guide-ug-100-7070/juno-user-guide-ug-100-7070/fluidigm%3Afile

After pipetting of all the assays and samples on the IFC, place the 96.96 IFC into the Juno and run the following script: Load Mix 96.96 GE

16. Real-Time PCR of the 96.96 IFC (with the Biomark HD)
  To switch on the Biomark first press the “on” button on the left side of the machine (for the PCR machine) and then press the green button "Computer Power" on the front of the Biomark (for the Computer inside of the Biomark).
  a. Double-click on the icon “BioMark Data Collection” on the desktop.
    ***Note:*** The lamp needs to be at −5°C before the run can start and it will only start the cooling as soon as the program “BioMark Data Collection” is opened (the cooling takes 5–10 min). Make sure that the status indicator for “Camera Temperature” is green (at −5.0°C).
  b. Click “Start a New Run”.
  c. Remove any dust particles from the IFC surface (use clear adhesive tape for that).
  d. Place the IFC in the Biomark HD (pay attention to the correct orientation, the barcode needs to face outward), and then click “Load”.
  e. Verify barcode and chip type, click “Next”.
  f. Select “This is a new chip run” or “Use a pre-defined Chip run”.
  g. Name the run and select a storage location.
  h. Click “Next”.
  i. Choose the application, passive reference, assay and probe:
    i Application: Gene Expression
    ii Passive reference: ROX
    iii Assay: Single probe
    iv Probes: EvaGreen
  j. Click “Next”
  k. Browse and select the following thermal protocol:
    **Biomark HD: GE 96**×**96 Fast PCR+Melt v2.pcl** (make sure that you are in the right folder “Protocols” > “GE”. If the protocol file is not visible, switch the settings of what files are displayed from “protocol files” to “all files”.)
  l. Make sure "Auto Exposure" is selected.
  m. Click “Next”.
  n. Verify the information, and then click “Start Run”.

### SNP typing of the mRNA with the 192.24 IFC using SNP Type Assays

**Timing: 5–6 h**

**Timing: 20–30 min for step 17**

**Timing: 5 min for step 18**

**Timing: 20–30 min for step 19**

**Timing: 40–50 min for step 20**

**Timing: 10 min for step 21**

**Timing: 60 min for step 22**

**Timing: The protocol “Load Mix 192.24 GT” takes roughly 28 min for step 23**

**Timing: 10 min + this program runs for roughly 80 min for step 24**

There are different formats for the SNP Typing analysis. We used the 192.24 IFC for SNP Typing (analyzing 24 SNPs in 192 cDNAs). For the SNP Typing analysis the SNP Type primers (ASP1/ASP2 and LSP) are employed (see Figures 1C and 1D).

The following steps were adapted from the Quick Reference Guide *Genotyping with the 192.24 IFC Using SNP Type Assays* (PN 100–3913 C1):

https://www.fluidigm.com/binaries/content/documents/fluidigm/resources/192.24-gt-snp%E2%80%90type-qr-100%E2%80%903913/192.24-gt-snp%E2%80%90type-qr-100%E2%80%903913/fluidigm%3Afile

Also refer to the *Biomark HD Data Collection* user guide (100–2451 Rev 14):

https://www.fluidigm.com/binaries/content/documents/fluidigm/resources/biomark-hd-data-collection-ug-100-2451/biomark-hd-data-collection-ug-100-2451/fluidigm%3Afile

The steps are written for the Instruments Juno and Biomark HD. When using different instruments, please refer to the guides listed above.

Fluidigm reagents should be thawed to 20°C–24°C, vortexed thoroughly and spun down prior to sample and assay mix preparation. Thaw and store the primer plates on ice and in the dark. Thoroughly vortex the primer plates and spin them down (1000 g / 1 min), prior to use. Frozen, non-Fluidigm reagents should be thawed and stored on ice, vortexed thoroughly and spun down prior to sample and assay mix preparation. Keep non-Fluidigm reagents and mixes containing non-Fluidigm reagents dark and on ice. The Fluidigm reagents used in this section are from the SNP Type™ 192.24 Genotyping Reagent Kit with Control Line Fluid (Fluidigm, Cat#: 100–4136).

Always include a water sample.

***Note:*** It is helpful to include gDNA of known genotype as a control during the SNP Typing of the mRNA.

**CRITICAL:** Vortex thoroughly and centrifuge all assay and sample solutions before pipetting into IFC inlets. Avoid bubbles.

17. Preparation of the SNP Type Assay Mixes
  ***Note:*** These prepared SNP Type Assay Mixes can be used for up to one year. Avoid multiple thaw-freeze cycles.
  a. In a DNA-free hood, prepare the SNP Type Assay Mixes by combining in each of 24 wells of a fresh 96 well plate (after vortexing and spinning of the reagents) (column 1–3):

**Table:** 

Reagent	Final concentration	Amount
SNP Type assay ASP1/ASP2 (100 μM each)	7.5 µM	1 μL
SNP Type assay LSP (100 μM each)	20 μM	2.67 μL
DNA Suspension Buffer (Teknova, Cat#: T0221)	N/A	9.67 μL
Total Volume	N/A	13.33 μL

18. Preparation of the Assay Pre-Mix
  a. Prepare in a fresh tube (after vortexing and spinning of the reagents):

**Table:** 

Combine:	Per well	26 assays
2× assay loading reagent (Fluidigm)	2.0 µL	52.0 µL
PCR-certified water	1.2 µL	31.2 µL
Total Volume	3.2 µL	83.2 µL

19. Preparation of the SNP Type Assay Plate (Primer Plate)
  ***Note:*** The SNP Type Assay Plate can be used for 3 weeks at –20°C or for up to 10 thaw-freeze cycles. So if multiple SNP type runs are planned prepare a SNP Type Assay Plate that can be used for multiple runs.
  a. Vortex the Assay Pre-Mix, spin it down and then add to each of 24 wells (column 1–3) of a new 96 well plate 3.2 μL of the Assay Pre-Mix (for 1 run - upscale as necessary).
  b. Transfer 0.8 μL (for 1 run – upscale as necessary) from the SNP Type Assay Mixes to the 24 wells of a 96 well plate containing already the Assay Pre-Mix with a 8-channel pipette.
  c. Seal the SNP Type Assay Plate with a qPCR foil.
  d. Vortex the plate thoroughly and spin it down (1000 g, 1 min).
  e. Store it on ice and in the dark.

**Pause point:** As the SNP Type Assay plate can be used for up to 3 weeks when stored at −20°C, the SNP Type Assay plate can be prepared in advance.

20. Preparation of the two Sample Plates (cDNA Plates)
  **CRITICAL:** Vortex thoroughly and centrifuge all substances.
  a. In a new tube combine for the Sample Mix:

**Table:** 

Reagent	Final concentration	Amount
Biotium Fast Probe Master Mix (Biotium, Cat#: 31005)	N/A	540 µL
20× SNPtype Sample Loading Reagent (Fluidigm)	N/A	54.0 µL
60× SNPtype reagent (Fluidigm)	N/A	18.0 µL
ROX Reference Dye (ThermoFisher Scientific, Cat#: 12223012)	N/A	6.48 µL
PCR-certified water	N/A	11.52 µL
**Total Volume**	N/A	630 µL
  b. Vortex the pipetted Sample Mix and centrifuge.
  c. Pipette 72 μL from the Sample Mix into each tube of a 8 PCR Stripe.
  d. Take two new 96 Well PCR Plates and aliquot 2.6 μL of the Sample Mix from the 8 PCR Stripe into each well of two 96 Well PCR Plate (Sample Plate) by using a 8-channel pipette.
  e. Transfer 1.9 μL of cDNA from two plates with pooled cDNA to the Sample Mix. Make sure to keep the layout of the original plates.
  f. Seal the Sample Plates with a qPCR foil.
  g. Vortex the plates thoroughly (make sure to vortex the entire plate) and spin them down (1000 g, 1 min).
  h. Store them on ice and in the dark.

**Figure 5:**
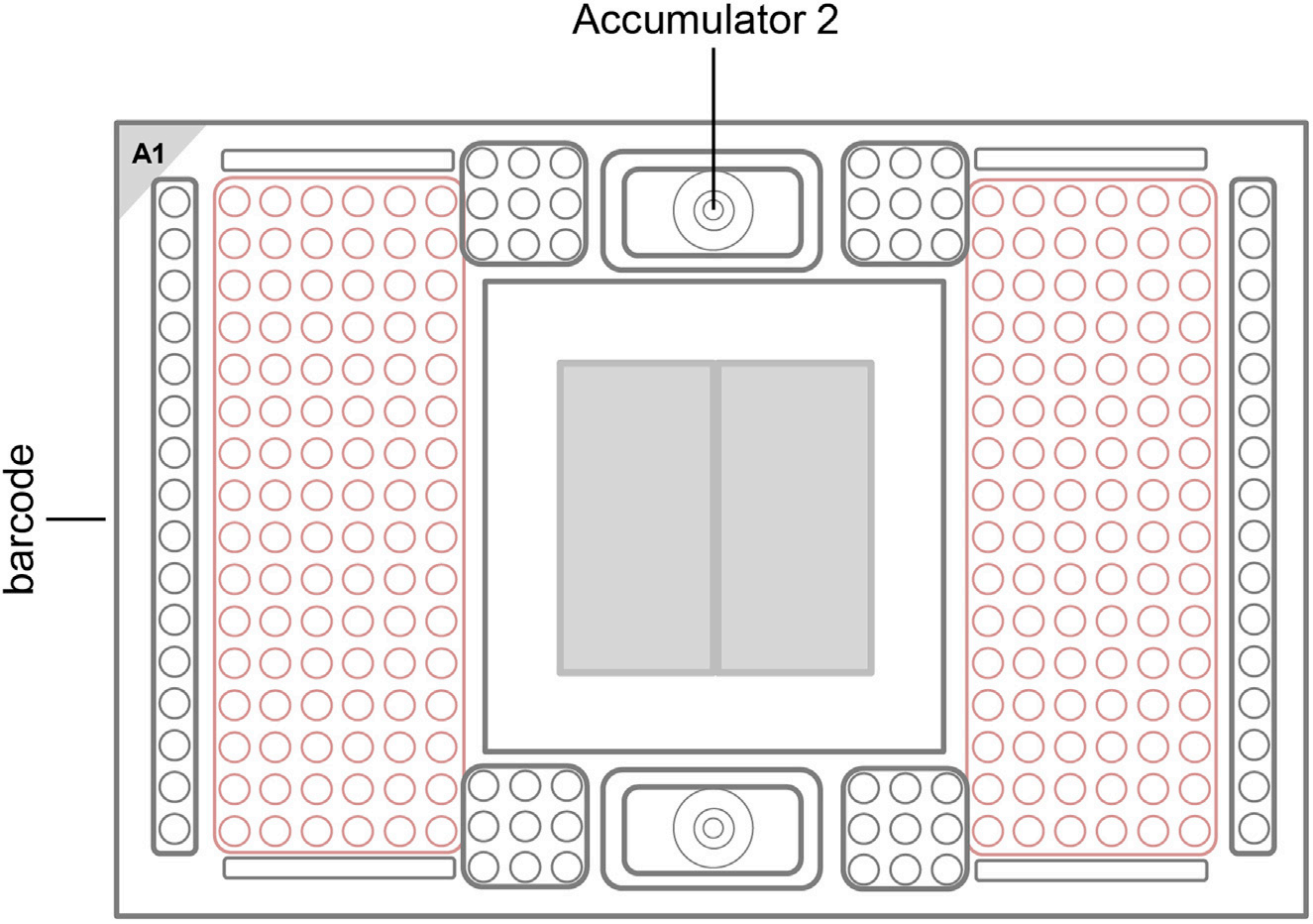
Layout of the 192.24 SNP Typing IFC, highlighting the position of the Accumulator 2 The Figure was recreated based on information and figures from the Quick Reference Guide *Genotyping with the 192.24 IFC Using SNP Type Assays* (PN 100-3913 C1): https://www.fluidigm.com/binaries/content/documents/fluidigm/resources/192.24-gt-snp%E2%80%90type-qr-100%E2%80%903913/192.24-gt-snp%E2%80%90type-qr-100%E2%80%903913/fluidigm%3Afile
  d. Take the 192.24 IFC, put it into a horizontal orientation and tilt it 45°.
  e. Using the syringe press down the valve of Accumulator 2.
  f. Visually confirm that the valve is open.
  g. Inject the control line fluid into the IFC by slowly pushing down the plunger of the syringe.
  h. After completely emptying the syringe, wait a few seconds before removing the syringe.
  i. It is normal that there is some liquid remaining in the syringe.
  j. Verify that the O-ring returns to its normal position after syringe removal.
  k. Make sure that no liquid was spilled.

**Figure 6:**
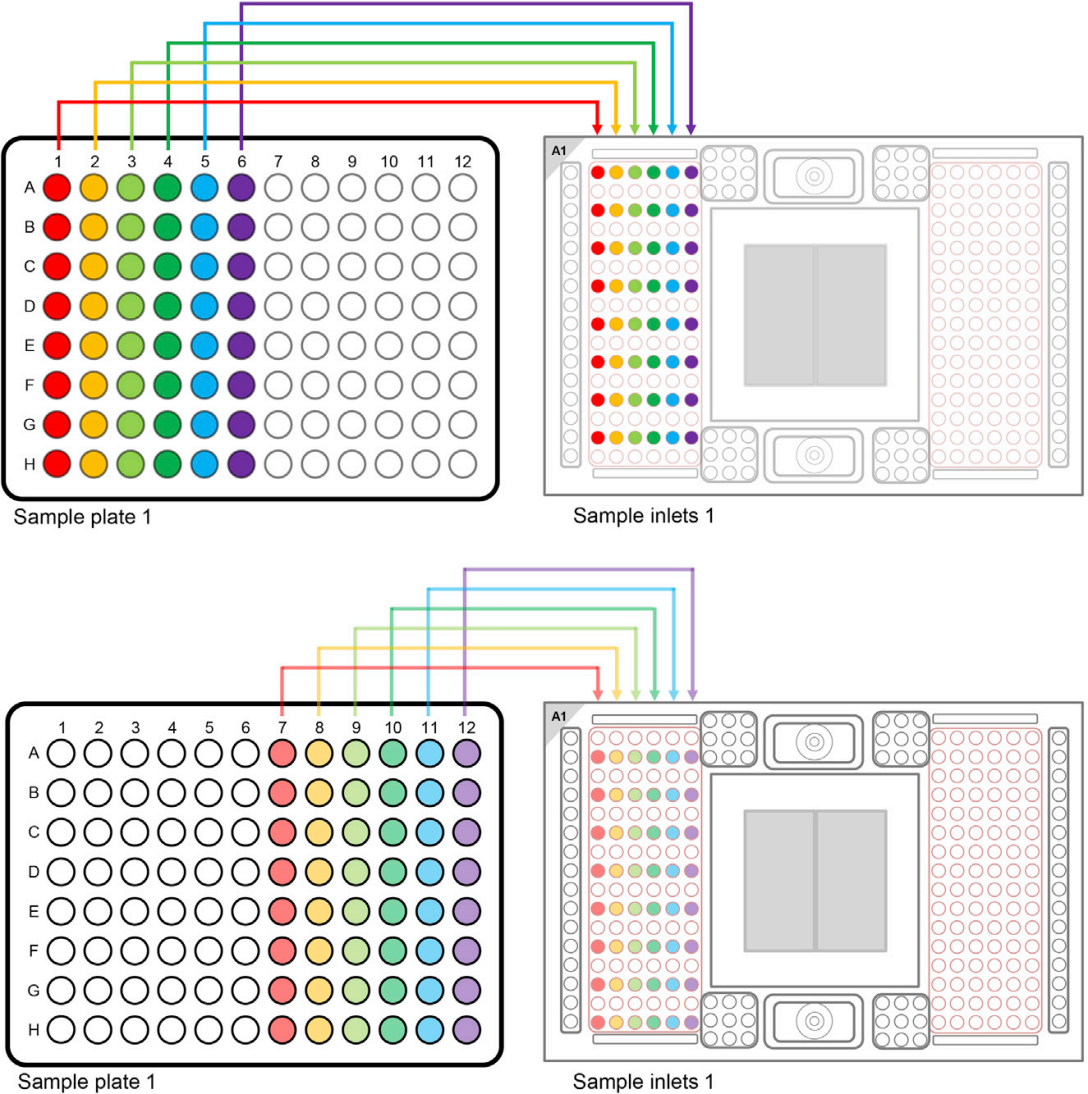
Pipetting Scheme for transferring Sample Plate 1 to the 192.24 SNP Typing IFC The Figure was recreated based on information and figures from the Quick Reference Guide *Genotyping with the 192.24 IFC Using SNP Type Assays* (PN 100-3913 C1): https://www.fluidigm.com/binaries/content/documents/fluidigm/resources/192.24-gt-snp%E2%80%90type-qr-100%E2%80%903913/192.24-gt-snp%E2%80%90type-qr-100%E2%80%903913/fluidigm%3Afile and the protocol *Using C1 to Capture Cells from Cell Culture and Perform Preamplification Using Delta Gene Assays* (PN 100-4904 L1): https://www.fluidigm.com/binaries/content/documents/fluidigm/resources/c1-delta%E2%80%90gene-pr-100%E2%80%904904/c1-delta%E2%80%90gene-pr-100%E2%80%904904/fluidigm%3Afile

**Figure 7:**
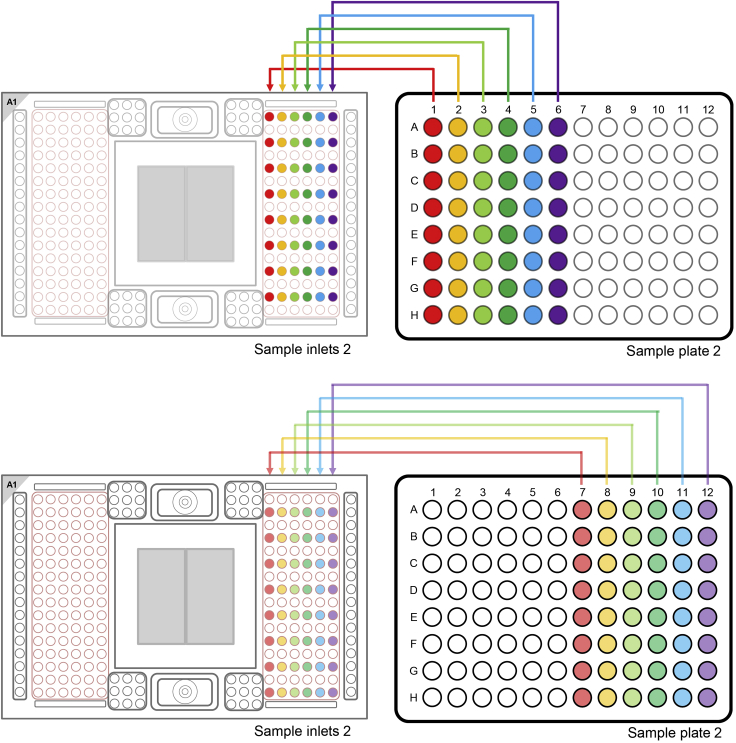
Pipetting Scheme for transferring Sample Plate 2 to the 192.24 SNP Typing IFC The Figure was recreated based on information and figures from the Quick Reference Guide *Genotyping with the 192.24 IFC Using SNP Type Assays* (PN 100-3913 C1): https://www.fluidigm.com/binaries/content/documents/fluidigm/resources/192.24-gt-snp%E2%80%90type-qr-100%E2%80%903913/192.24-gt-snp%E2%80%90type-qr-100%E2%80%903913/fluidigm%3Afile and the protocol *Using C1 to Capture Cells from Cell Culture and Perform Preamplification Using Delta Gene Assays* (PN 100-4904 L1): https://www.fluidigm.com/binaries/content/documents/fluidigm/resources/c1-delta%E2%80%90gene-pr-100%E2%80%904904/c1-delta%E2%80%90gene-pr-100%E2%80%904904/fluidigm%3Afile
  c. Vortex the assay plate thoroughly and spin down (1 min / 1000 g), before pipetting 3 μL with an 8-channel pipette from each assay from the SNP Type Assay Plate on the 192.24 IFC (see Figure 8 for the IFC Pipetting Map).

21. Prepare the 192.24 IFC
  The following steps were adapted from *Control Line Fluid Loading Procedure* (PN 68000132 r07):
  https://www.fluidigm.com/binaries/content/documents/fluidigm/resources/control-line-fluid-loading-procedure-68000132-qr/control-line-fluid-loading-procedure-68000132-qr/fluidigm%3Afile
  **CRITICAL:** The control line fluid inhibits the read-out of the data, so make sure, that it does not drop on the IFC. If the control line fluid was spilled on the IFC, try to carefully remove it using 70% Ethanol.
  **CRITICAL:** Wear safety goggles while applying the control line fluid.
  a. Before opening the 192.24 IFC, evacuate carefully the air out of the syringe.
  b. Open the package of the 192.24 IFC.
  c. Using a 100 μL pipette tip make sure that the top valve (when the bar code of the IFC is on the left, see Figure 5) (Accumulator 2) opens easily.

22. Loading the 192.24 IFC
  After the addition of the control line fluid, pipette the assays and the samples on the 192.24 IFC.
  **CRITICAL:** Vortex the SNP Type Assay Plate and the Sample Plates thoroughly and spin down before loading them on the 192.24 IFC.
  **CRITICAL:** Always halt at the first stop of the pipette when pipetting. Try to avoid bubbles for every liquid that you add to the IFCs. Use a hollow needle (cannula - e.g. 0.8 mm diameter) to destroy bigger bubbles.
  Do not leave any inlets empty.
  a. Vortex the Sample Plate thoroughly and spin down (1 min / 1000 g) before loading them on the 192.24 IFC.
  b. Pipette 3 μL with an 8-channel pipette of each sample from the two Sample Plates on the 192.24 IFC (see Figures 6 and 7).

**Figure 8:**
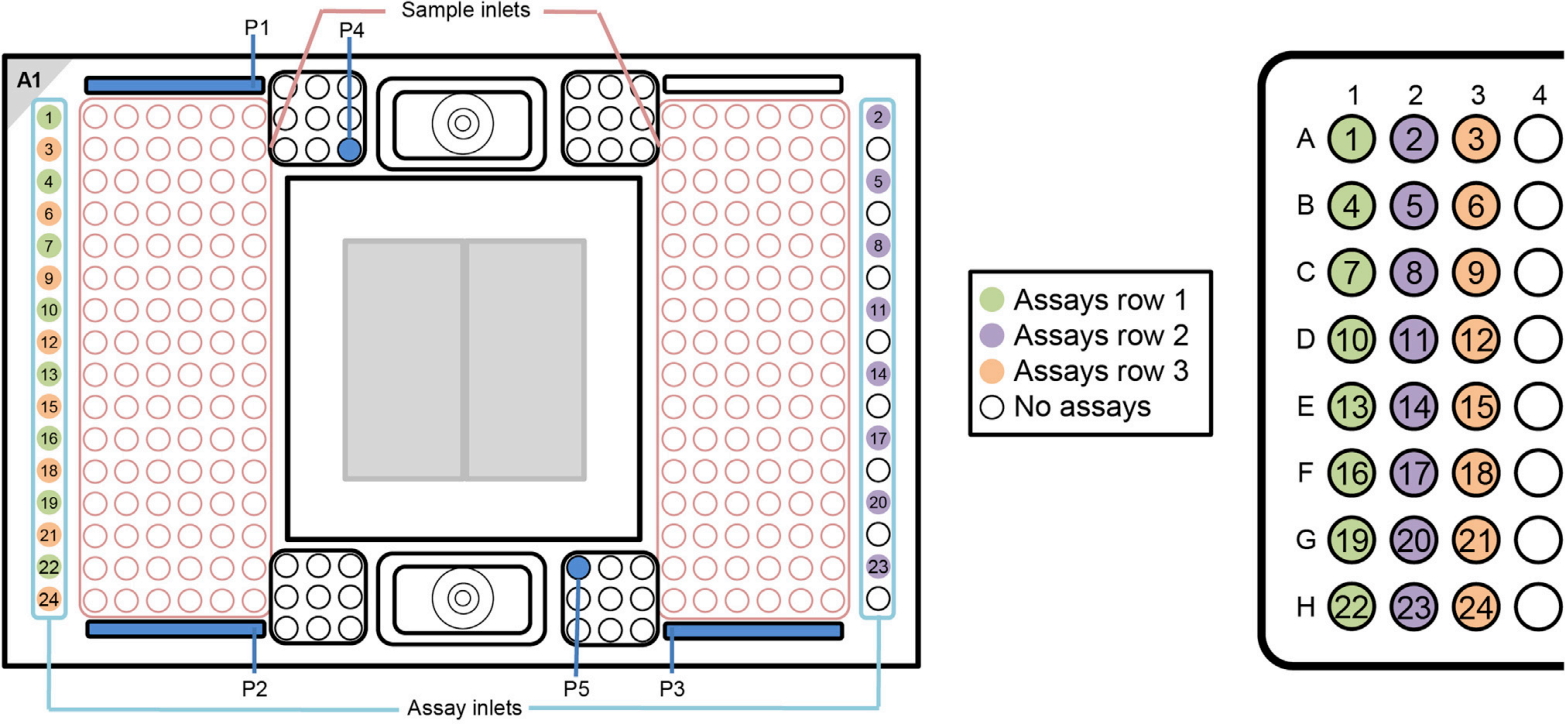
Pipetting Scheme for adding the assays and the pressure fluid to the 192.24 SNP Typing IFC The Figure was recreated based on information and figures from the Quick Reference Guide *Genotyping with the 192.24 IFC Using SNP Type Assays* (PN 100-3913 C1): https://www.fluidigm.com/binaries/content/documents/fluidigm/resources/192.24-gt-snp%E2%80%90type-qr-100%E2%80%903913/192.24-gt-snp%E2%80%90type-qr-100%E2%80%903913/fluidigm%3Afile
  d. Pipette 150 μL of pressure fluid into the inlets P1, P2 and P3 (see Figure 8).
  e. Pipette 20 μL of pressure fluid into the inlets P4 and P5 (see Figure 8).
  f. Do not leave any wells empty.
  g. To every unused assay inlet, add 3.2 μL Assay Pre-Mix and 0.8 μL DNA-free water.
  h. To every unused sample inlet, add 2.6 μL of Sample Mix and 1.9 μL of DNA-free water.
  i. Check for bubbles and destroy them using a pipette tip or hollow needle / cannula.
  j. Use a fresh pipette tip / cannula for every well.

23. Load Mix of the 192.24 IFC

***Note:*** Start the IFC run within 1 h of adding the assays and samples on the IFC.

Depending on the layout of the IFC (96.96 or 192.24) the Juno needs a different interface plate. Refer to p. 27ff of the *Juno System* user guide (PN 100–7070 r12) on how to change the interface plate:

https://www.fluidigm.com/binaries/content/documents/fluidigm/resources/juno-user-guide-ug-100-7070/juno-user-guide-ug-100-7070/fluidigm%3Afile

After pipetting of all the samples, assays and pressure fluid, place the 192.24 IFC into the Juno and run the following script: Load Mix 192.24 GT.

24. Thermal-Cycling of the 192.24 IFC (with the Biomark HD)
  To switch on the Biomark first press the “on” button on the left side of the machine (for the PCR machine) and then press the green button "Computer Power" on the front of the Biomark (for the Computer inside of the Biomark).
  a. Double-click on the icon “BioMark Data Collection” on the desktop.
    ***Note:*** The lamp needs to be at −5°C before the run can start and it will only start the cooling as soon as the program “BioMark Data Collection” is opened (the cooling takes 5–10 min). Make sure that status indicator for “Camera Temperature” is green (at −5.0°C).
  b. Click “Start a New Run”.
  c. Remove any dust particles or debris from the IFC surface (use clear adhesive tape for that).
  d. Place the IFC in the Biomark HD (pay attention to the correct orientation, the barcode needs to face outward), and then click “Load”.
  e. Verify barcode and chip type, click “Next”.
  f. Select “This is a new chip run” or “Use a pre-defined Chip run”.
  g. Name the run and select a storage location.
  h. Click “Next”.
  i. Choose the application, passive reference, and probes:
    i Application: Genotyping
    ii Passive reference: ROX
    iii Select probes manually:
    Probe 1: SNPtype-FAM
    Probe 2: SNPtype-HEX
  j. Click “Next”.
  k. Browse and select the following thermal protocol:
    **SNPtype 192.24 v1** (make sure that you are in the right folder “Protocols” > “GT”. If the protocol file is not visible, switch the settings of what files are displayed from “protocol files” to “all files”.)
  l. Make sure "Auto Exposure" is selected.
  m. Click “Next”.
  n. Verify the information, and then click “Start Run”.

### Quantification and data processing

Graphs were generated with GraphPad Prism 9 and schemes were created with PowerPoint 2016. Statistical analysis was done with GraphPad Prism 9. Mann-Whitney test was used for comparison between two unpaired groups.

The gene expression data can be analyzed with the Fluidigm Real-Time PCR Analysis software and the SNP Typing data can be analyzed with the Fluidigm SNP Genotyping Analysis software. The softwares can be downloaded here: https://www.fluidigm.com/software.

For a step-by-step description of how to analyze the gene expression data with the Fluidigm Real-Time PCR Analysis software refer to p. 19ff of the *Real-Time PCR Analysis* user guide (PN 68000088 Rev 16):

https://www.fluidigm.com/binaries/content/documents/fluidigm/resources/real-time-pcr-analysis-ug-68000088/real-time-pcr-analysis-ug-68000088/fluidigm%3Afile

For a step-by-step description of how to analyze SNP Typing data with the SNP Genotyping Analysis Software refer to p. 29ff of the *SNP Genotyping* user guide (PN 68000098 Rev.18):

https://www.fluidigm.com/binaries/content/documents/fluidigm/resources/snp-gt-analysis-ug-68000098/snp-gt-analysis-ug-68000098/fluidigm%3Afile

Capture sites not containing a single, individual cell have already been excluded from further analysis by pooling of the cDNA, if not, exclude them now. As an additional control step the expression of reference genes were employed to verify that a normal, live cell was processed. Next to reference genes that are generally employed in quantitative PCR (Vandesompele et al., 2002), it is also possible to employ genes that have a high expression in the target cells. E.g. in pDCs single cell expression of HLA-DR would be a possible target gene. Individual cells without detectable mRNA expression in two out of three reference or target genes (*B2M, RPL13A, GAPDH*) were excluded from further analysis.

In most qPCR samples the results are normalized to one or more reference genes to control for variations on factors such as extraction or reverse-transcription yield (Bustin et al., 2009). However, in a standardized setting working with single cells, normalization is not necessary which has been discussed previously (McDavid et al., 2013; Arsenio et al., 2014). The Real-Time PCR Analysis software assigns the value of 999 to reactions without detectable mRNA. As suggested by Litvak et al. a limit of detection (LOD) of a ct value of 24 was assigned to every ct value exceeding 24 and the mRNA expression is defined as 2^(LOD-ct)^ (Livak et al., 2013). Hence an expression value of 1 specifies a cell without detectable mRNA expression in the analyzed gene. This way of analyzing the data has recently been published (Hipp et al., 2017; Hess et al., 2020).

To control the gene expression data in the Fluidigm Real-Time PCR Analysis Software the Melting Curve peaks are analyzed. A range of +/- 1.2°C was allowed to take into account amplification of the regions containing either the minor or the major allele. When analyzing regions not containing different alleles it is possible to adapt the temperature range. The median of the temperature peak was determined from all non-failed reactions of one gene. The other settings for melting curve analysis (Peak Sensitivity: 7; Peak Ratio Threshold: 0.8) were left unchanged in the Fluidigm Real-Time PCR Analysis Software. For all reactions that were flagged as failed under these settings the ct value was assigned to the LOD.

For the SNP Typing of the mRNA, sometimes individual cells are located in between the clear clusters. Here it is possible to rerun the SNP Typing of the mRNA or to assign them as “no detectable mRNA”. If there are no clear clusters of the populations, refer to *Problem 4* below. ASP1 and ASP2 are the primers that differentiate between the minor and the major allele. In the factory setting the results for ASP1 are displayed on the x axis, whereas the results for ASP2 will be shown on the y axis. Which primer represents which nucleotide can be found in the design file that is supplied by Fluidigm.

## Expected outcomes

**Figure 9:**
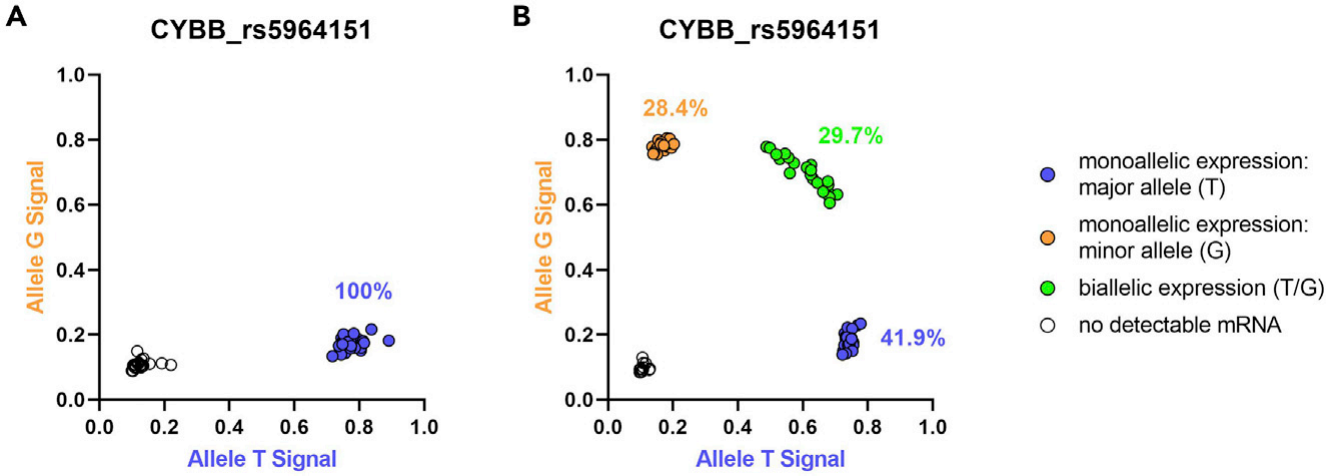
Investigation of escape of *CYBB* from XCI in human pDCs Unstimulated pDCs derived from frozen PBMCs of one female individual are displayed. Each dot represents one single cell. Blue circles are pDCs with monoallelic expression of the major allele, orange dots are pDCs with monoallelic expression of the minor allele, and green circles represent pDCs with biallelic expression (major and minor allele) in the same cell, reflecting escape from XCI. Black circles represent cells without any detectable mRNA. Percentages were calculated using all cells with detectable mRNA. Expression patterns were determined using the SNP rs5964151 in *CYBB*. (A) SNP typing of the mRNA of one female homozygous for the major allele of the SNP rs5964151 in *CYBB*. (B) SNP typing of the mRNA of one female heterozygous for the SNP rs5964151, demonstrating escape of *CYBB* from XCI.

This protocol describes a SNP based approach to investigate the expression pattern of a gene at the single-cell level and thus determine the possible escape from XCI in human, single pDCs. The expression pattern of the gene(s) can then be linked to the mRNA expression of up to 200 mRNAs of these individual cells. It is estimated that 10%–25% of genes on the X chromosome escape from XCI (Carrel and Willard, 2005) with suggested heterogeneity of escape from XCI between different individuals and tissues (Cotton et al., 2013; Tukiainen et al., 2017). As a control, SNP typing of the mRNA is performed on a female individual homozygous for the major allele of SNP rs5964151 in the gene *CYBB* to demonstrate only monoallelic expression of single pDCs (Figure 9A). In a female heterozygous for the SNP rs5964151, the escape plot demonstrates escape of *CYBB* from XCI in 29.7% of the pDCs of that female individual (Figure 9B).

**Figure 10:**
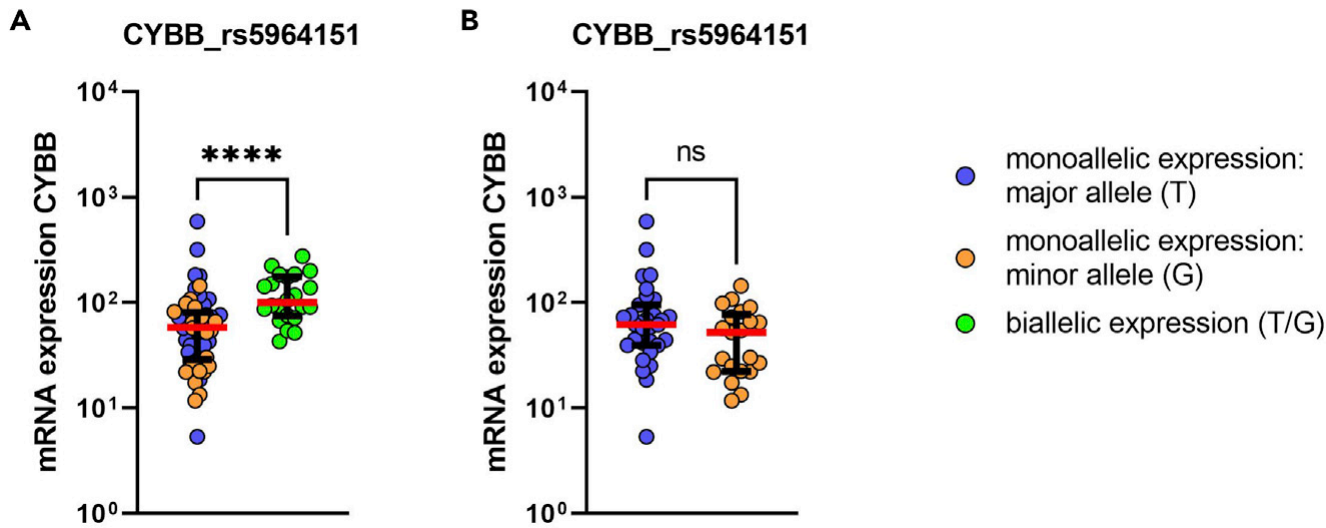
The influence of escape of *CYBB* from XCI and the influence of the SNP rs5964151 in human pDCs on the mRNA expression of CYBB at the single-cell level (A) Comparison of the mRNA expression of CYBB between female pDCs with monoallelic expression of CYBB (blue circles monoallelic expression of the major allele, orange circles monoallelic expression of the minor allele) to female pDCs with biallelic expression (pDCs with escape from XCI, green circles). Unstimulated pDCs derived from frozen PBMCs of one female are displayed. Each dot represents one single cell. Median (red bar) with interquartile range (black bars) is shown. Mann–Whitney test was used for statistical analysis. ∗∗∗∗p < 0.0001. Expression patterns were determined using the SNP rs5964151 in *CYBB*. (B) Comparison of the mRNA expression of CYBB between female pDCs with monoallelic expression of the major allele (blue circles) and monoallelic expression of the minor allele (orange circles). Unstimulated pDCs derived from frozen PBMCs of one female are displayed. Each dot represents one single cell. Median (red bar) with interquartile range (black bars) is shown. Mann–Whitney test was used for statistical analysis. ns = not significant.

Following the expression analysis of genes at the single-cell level (monoallelic expression of the major allele / monoallelic expression of the minor allele / biallelic expression (in case of X chromosomal genes, this means escape from XCI) / no expression), it is then possible to assess the mRNA expression of that gene and other genes at the single-cell level. pDCs with escape of *CYBB* from XCI show a significantly higher mRNA expression of CYBB compared to pDCs without escape (Figure 10A). In addition, it is possible to investigate the possible influence of the SNP at the single-cell level, as a nucleotide exchange in the 3′UTR could have an influence on the transcription level of that gene, for example due to a different binding affinity of a regulating microRNA to the mRNA (Hunt et al., 2014). Comparing the major (T) to the minor allele (G) of the SNP rs5964151 in *CYBB* on the gene expression of CYBB, no significant difference in mRNA expression of CYBB was observed (Figure 10B).

## Limitations

A limitation of this SNP based protocol is that escape from XCI can only be detected in genes that have a suitable SNP in the mature mRNA of the respective gene and it is necessary to use cells of a female individual who is heterozygous for the respective SNP. There are other possibilities of detecting escape from XCI. Souyris et al. used fluorescence in situ hybridization (FISH) to visualize the inactive X chromosome by targeting the XIST RNA thus confirming TLR7 transcription from the inactivated X chromosome and escape from XCI in female, human B cells (Souyris et al., 2018). However, with the RNA FISH approach quantification of the mRNA expression would currently be a lot more challenging.

## Troubleshooting

### Problem 1

Low capture rate. See step *Using the C1 platform to generate pre-amplified cDNA of individual captured cells > 5. Imaging of the cells.*

### Potential solution

The capture rate in the C1 IFC is dependent on the buoyancy of the cells in solution, the cell concentration and the size of the IFCs that is used. The buoyancy is controlled by the ratio of cell suspension to Suspension Reagent (Fluidigm). When you visualize the cells prior to the loading step and the cells are not equally distributed throughout the cell suspension reevaluate the ratio of Suspension Reagent as described above (*Adjusting the buoyancy of your cell type of interest).*

If there are a lot of empty capture sites, the size of the capture sites might be too small and if there are multiple capture sites with more than one cell, the size of the capture sites might be too big. Reevaluate the size of the cells as described above (*Determine the size your cell type of interest).*

If the majority of capture sites are empty, increase the cell concentration for the loading step. Sorting directly into the cell loading inlet gives the highest control over the cell number.

### Problem 2

There are multiple melting curves for the amplification of a gene. See steps *Gene Expression of pre-amplified samples with the 96.96 IFC using Delta Gene Assays > 16. Real-Time PCR of the 96.96 IFC (with the Biomark HD)* and *Quantification and data processing*.

### Potential solution

**Figure 11:**
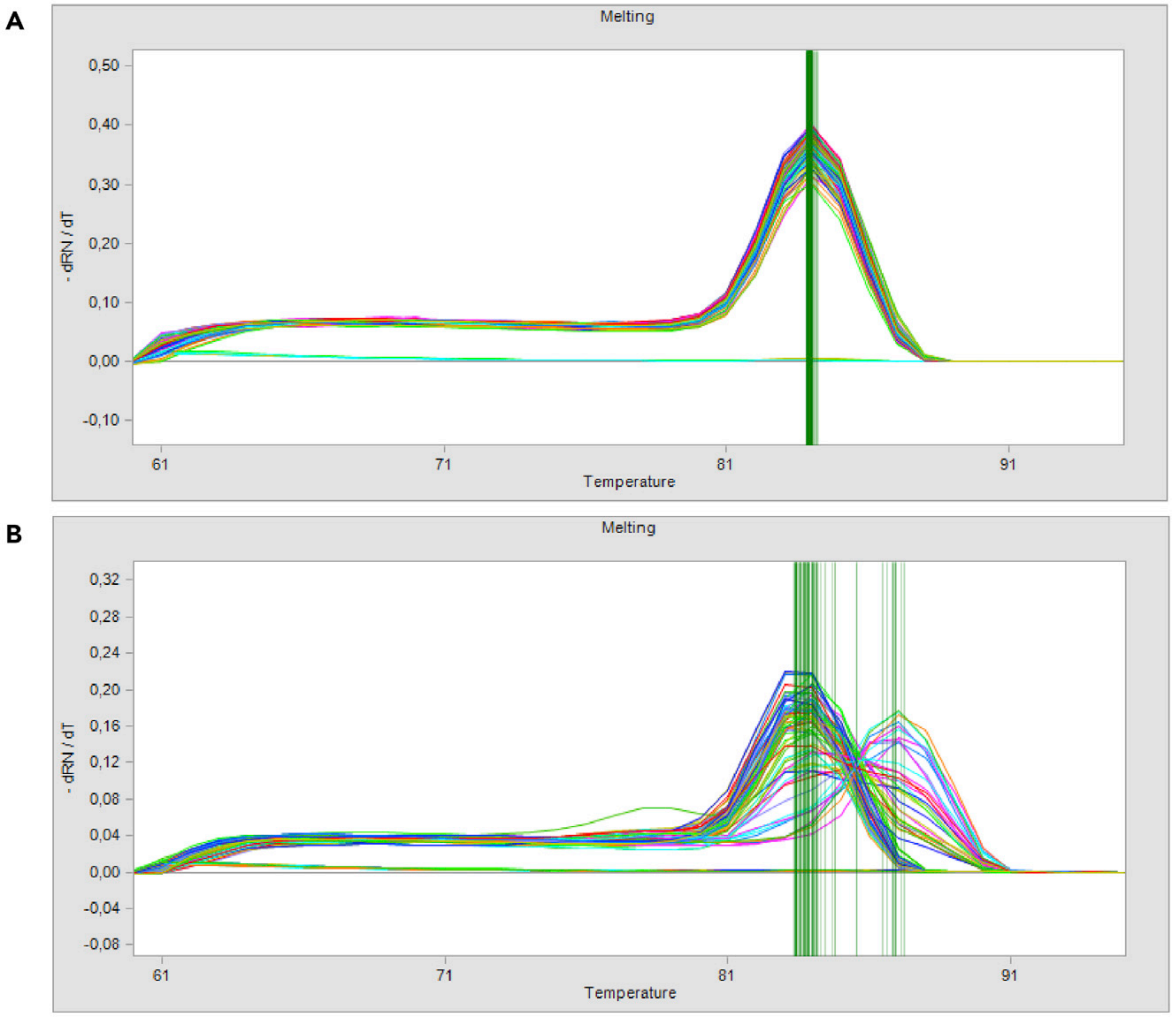
Screenshots of melting curves that were taken in the Fluidigm Real-Time PCR Analysis Software (A) Similar melting curves of the gene B2M in single, human pDCs. (B) Problematic melting curves displaying multiple peaks of the gene IRF2BP2 in single, human pDCs.

The melting curves for the same gene should have the same peak with a similar curve (s. Figure 11A). If this is not the case, set the ct value to the LOD for the individual cell for the specific gene as outlined in *Quantification and data processing,* since a different melting curve peak indicates a different or unspecific amplicon. However if there are multiple melting curve peaks with a similar distribution between the individual cells, the primers used for this gene should be redesigned.

### Problem 3

There is no clear clustering of the populations for the SNP Typing of the gDNA. See steps *Isolation of gDNA and SNP typing of Cohort > 5. SNP Typing of the gDNA* and *Quantification and data processing*.

### Potential solution

Make sure to perform a STA prior to the SNP Typing of the gDNA as this increases the quality of the clusters and the results (refer to the section *Specific Target amplification (STA) of gDNA).*

If the sequences for designing of the SNP Type primers were added manually, make sure that the sequence and the nucleotides for the major and minor allele were annotated correctly.

If the STA does not improve the results, redesigning the primers is another option (even though the primer design is limited by the location of the SNP in the mRNA) or to research whether there is a different SNP in the mature mRNA that could be used. If there are no clear clusters with the gDNA, this will most likely also lead to unclear clustering of the mRNA (see also *Problem 4*).

### Problem 4

There is no clear clustering of the populations for the SNP Typing of the mRNA. See steps *SNP Typing of the mRNA with the 192.24 IFC using SNP Type Assays > 24. Thermal-Cycling of the 192.24 IFC (with the Biomark HD)* and *Quantification and data processing*.

### Potential solution

If there is clear clustering with the primers when performing SNP Typing of gDNA, then the number of PCR cycles could be adjusted for the SNP Typing of the mRNA. If the results all cluster together in the location of the biallelic cells, reduce the number of PCR cycles (which will likely be the case for genes with a very high mRNA expression level). If the SNP typing of the gDNA does not generate clear clusters, then redesign the primers or investigate whether there is a different suitable SNP.

To adjust a thermocycling protocol of the Biomark perform the following steps:

Open the software Biomark Data Collection
Go to tools > protocol editor > new > protocol type
Choose protocol type and select the different options
Cycle numbers, times and temperature can be edited manually

Also see page 58ff of the *Biomark HD Data Collection* user guide (100–2451 Rev 14):

https://www.fluidigm.com/binaries/content/documents/fluidigm/resources/biomark-hd-data-collection-ug-100-2451/biomark-hd-data-collection-ug-100-2451/fluidigm%3Afile

For rs3853839 (*TLR7*), rs7051161 (*RPS6KA3*), rs5964151 (*CYBB*), rs700 (*BTK*), rs2495636 (*IL13RA1*) no adjustment of the cycle number in human pDCs was necessary (Hagen et al., 2020).

### Problem 5

Positive signal for the negative Control (Tube Control). See step *Using the C1 platform to generate pre-amplified cDNA of individual captured cells > 7. Negative and positive Control* and *Quantification and data processing*.

### Potential solution

Since performing the negative Control (Tube Control) alongside, but outside of the Fluidigm IFC with a lot of PCR cycles, there is the possibility of receiving a positive signal for the tube control that is due to e.g., the tubes used for the preparation of the negative control, but does not indicate a contamination of the reagents used for the C1 run. As the LOD is defined as a ct value of 24, only a lower ct value than 24 is critical (refer to the section *Quantification and data processing*). As an alternative control, it is possible to use an empty capture site from the C1 run as a negative control. An empty capture site that did not generate an mRNA signal for a specific primer pair, demonstrates that there was no unspecific amplification due to a contamination (e.g., in one of the mixes). However, the negative control (tube control) should still always be included, as it is not guaranteed that a C1 run will produce an empty capture site.

To minimize the risk of contaminations prepare single-use aliquots as outlined in the section *Aliquot Preparation*.

## Resource availability

### Lead contact

Further information and requests for resources and reagents should be directed to and will be fulfilled by the lead contact, Marcus Altfeld (marcus.altfeld@leibniz-hpi.de).

### Materials availability

This study did not generate new unique reagents.

### Data and code availability

This study did not generate/analyze datasets or code.
